# Lysin (K)-specific demethylase 1 inhibition enhances proteasome inhibitor response and overcomes drug resistance in multiple myeloma

**DOI:** 10.1186/s40164-023-00434-x

**Published:** 2023-08-10

**Authors:** Cecilia Bandini, Elisabetta Mereu, Tina Paradzik, Maria Labrador, Monica Maccagno, Michela Cumerlato, Federico Oreglia, Lorenzo Prever, Veronica Manicardi, Elisa Taiana, Domenica Ronchetti, Mattia D’Agostino, Francesca Gay, Alessandra Larocca, Lenka Besse, Giorgio Roberto Merlo, Emilio Hirsch, Alessia Ciarrocchi, Giorgio Inghirami, Antonino Neri, Roberto Piva

**Affiliations:** 1 Department of Molecular Biotechnology and Health Sciences, University of Turin, Turin, Italy; 2 Department of Physical Chemistry, Rudjer Boskovic Insitute, Zagreb, Croatia; 3 Laboratory of Translational Research, Azienda USL-IRCCS Reggio Emilia, Reggio Emilia, Italy; 4 Hematology, Fondazione Cà Granda IRCCS Policlinico, Milan, Italy; 5 Department of Oncology and Hemato-Oncology, University of Milan, Milan, Italy; 6 Città Della Salute e della Scienza Hospital, Turin, Italy; 7 Experimental Oncology and Hematology, Department of Oncology and Hematology, St. Gallen Cantonal Hospital, St. Gallen, Switzerland; 8 Department of Biology, Faculty of Medicine, Masaryk University, Brno, Czech Republic; 9 Department of Pathology and Laboratory Medicine, Weill Cornell Medicine, New York, NY USA; 10 Scientific Directorate, Azienda-USL IRCCS di Reggio Emilia, Reggio Emilia, Italy

**Keywords:** Multiple myeloma, B-cell neoplasms, Proteasome inhibitors, Synthetic lethality, LSD1, Drug resistance

## Abstract

**Background:**

Multiple myeloma (MM) is an incurable plasma cell malignancy, accounting for approximately 1% of all cancers. Despite recent advances in the treatment of MM, due to the introduction of proteasome inhibitors (PIs) such as bortezomib (BTZ) and carfilzomib (CFZ), relapses and disease progression remain common. Therefore, a major challenge is the development of novel therapeutic approaches to overcome drug resistance, improve patient outcomes, and broaden PIs applicability to other pathologies.

**Methods:**

We performed genetic and drug screens to identify new synthetic lethal partners to PIs, and validated candidates in PI-sensitive and -resistant MM cells. We also tested best synthetic lethal interactions in other B-cell malignancies, such as mantle cell, Burkitt’s and diffuse large B-cell lymphomas. We evaluated the toxicity of combination treatments in normal peripheral blood mononuclear cells (PBMCs) and bone marrow stromal cells (BMSCs). We confirmed the combo treatment’ synergistic effects ex vivo in primary CD138+ cells from MM patients, and in different MM xenograft models. We exploited RNA-sequencing and Reverse-Phase Protein Arrays (RPPA) to investigate the molecular mechanisms of the synergy.

**Results:**

We identified lysine (K)-specific demethylase 1 (LSD1) as a top candidate whose inhibition can synergize with CFZ treatment. LSD1 silencing enhanced CFZ sensitivity in both PI-resistant and -sensitive MM cells, resulting in increased tumor cell death. Several LSD1 inhibitors (SP2509, SP2577, and CC-90011) triggered synergistic cytotoxicity in combination with different PIs in MM and other B-cell neoplasms. CFZ/SP2509 treatment exhibited a favorable cytotoxicity profile toward PBMCs and BMSCs. We confirmed the clinical potential of LSD1-proteasome inhibition in primary CD138+ cells of MM patients, and in MM xenograft models, leading to the inhibition of tumor progression. DNA damage response (DDR) and proliferation machinery were the most affected pathways by CFZ/SP2509 combo treatment, responsible for the anti-tumoral effects.

**Conclusions:**

The present study preclinically demonstrated that LSD1 inhibition could provide a valuable strategy to enhance PI sensitivity and overcome drug resistance in MM patients and that this combination might be exploited for the treatment of other B-cell malignancies, thus extending the therapeutic impact of the project.

**Supplementary Information:**

The online version contains supplementary material available at 10.1186/s40164-023-00434-x.

## Introduction

The development of proteasome inhibitors (PIs) for the treatment of multiple myeloma (MM) patients, including those with relapsed/refractory disease, has markedly prolonged overall survival in the last decades [1, 2]. Moreover, clinical trials are underway to evaluate their efficacy against other tumors and for noncancer applications (https://clinicaltrials.gov) [3]. MM cells are especially sensitive to the proteasome inhibition due of their heightened need to eliminate disrupted proteins, maintain homeostasis, and support cell proliferation [4, 5]. Inhibition of proteasome leads to accumulation of misfolded proteins, enhancement of proteotoxic stress, endoplasmic reticulum stress, activation of unfolded-protein response (UPR), DNA-damage responses (DDR), cell cycle arrest, and subsequent apoptosis [4]. Despite clinical benefits of PIs, most patients experience drug resistance [6], or suffer severe side effects such as cardiovascular toxicity and neuropathy [7]. Several mechanisms of PI resistance have been identified including deregulation of components of the ubiquitin proteasome system, mutations in the proteasome subunit β type 5 (PSMB5) of the 20S proteasome, autophagy induction, up-regulation of the antioxidant response, and down-regulation of protein synthesis [8, 9].

MM is a highly complex disease with substantial intra-clonal genetic heterogeneity [10], which makes challenging to find effective and personalized therapeutic regimens. Great efforts are making in informing on possible treatment options, by direct analysis of patients biopsies ex vivo, using “omics” techniques [11]. Despite the genetic nature of the disease, it is increasingly recognized that epigenetic machinery plays a crucial role in MM, contributing to the high plasticity of myeloma cells and the development of therapy resistance [12–14]. Recent findings have revealed transcriptional and epigenetic mechanisms of drug resistance that may lead to the emergence of reversible drug-tolerant cells [15–17]. Accordingly, several histone modification and chromatin remodeling factors, such as Enhancer of Zeste 2 Polycomb Repressive Complex 2 Subunit (EZH2), Lysine Demethylase 5 and 6 (KDM5, KDM6), and Histone Deacetylases (HDACs) have been demonstrated to be tolerance-related genes, deregulated in cancer cells after therapeutic regimens [15, 18–20]. Accordingly, refractory disease can be reversed by epigenetic reprogramming, exploiting combination and intermittent therapies. Besides, cell metabolism adaptation, cell identity changes, and microenvironment hijacking might also participate in cancer cell plasticity [17]. Consistently, we showed that the inhibition of the metabolic enzyme isocitrate dehydrogenase 2 (IDH2) is an effective strategy to restore PIs sensitivity in MM and other B-cell malignancies [21]. All these data highlight the importance of preventing the emergence of treatment-induced drug-tolerant cells in cancer therapy. Combinatorial screenings using shRNA, CRISPR/Cas9, or drug libraries have been widely applied and are valuable options to identify effective synergistic drug combinations [19, 22–25].

Here, using genetic and pharmacologic loss-of-function screens we uncovered that the inhibition of the epigenetic eraser LSD1 strongly synergize with the PI carfilzomib. While the involvement of epigenetic factors in regulating PIs responsiveness has been previously described [23], the understanding of LSD1's functions in MM is still limited. Our findings suggest that LSD1 inhibition is a promising new therapeutic option for MM and other pathologies, potentially overcoming PIs resistance.

## Methods

Detailed experimental procedures for cell culture conditions, reagents, MM patients, healthy donors samples, virus production, in vitro transduction, LSD1 constructs, mutagenesis, generation of inducible cell lines, shRNA screening, library preparation, RNA-Sequencing, Reverse Phase Protein Array, purification of total RNA, Reverse Transcription-quantitative Polymerase Chain Reaction (RT-qPCR), western blotting, analysis of apoptosis, cell cycle, ATPlite assay, and zebrafish housing are included in supplementary methods.

### Drug screening

Primary screening was performed using a target selective inhibitor library (L3500-Selleck Chemicals https://www.selleckchem.com/screening/selective-library.html) assembled with 320 small molecule inhibitors covering 123 key targets implicated in a wide variety of signaling pathways. U266^PIR^ cells treatment was performed in duplicate using 4 concentrations (10 μM, 1 μM, 100 nM, and 10 nM). After 2 h cells were treated with a sublethal dose of Carfilzomib (2.5 nM) or with control DMSO. Cell viability was assessed using Cell TiterGlo (Promega) luminescence assay performed at day 0- and 72 h post-treatment, in duplicate. Growth Rate (GR) was calculated as the ratio between luminescence at day 0 and luminescence at day 3, normalized to DMSO-treated cells. Combined drug effect was determined by Excess over Bliss (EOB) analysis on GR value for all concentrations, according to the formula [26]:

$$\mathrm{EOB}=\left(1-\mathrm{GR }\left(\mathrm{combination}\right)\right)-\left(1-\mathrm{GR}\left(\mathrm{CFZ}\right)\right)-\left(1-\mathrm{GR}\left(\mathrm{drug}\right)\right)+\left(1-\mathrm{GR}\left(\mathrm{CFZ}\right)\right)\left(1-\mathrm{GR}\left(\mathrm{drug}\right)\right)$$

The most synergistic drugs (TOP15) were chosen posing an arbitrary cut-off of EOB > 0.2 in at least two concentrations and selected for subsequent analysis.

### Murine xenografts

KMS-28-TTA_shLSD1-D6 cells (5 × 10^5^) suspended in phosphate-buffered saline (PBS)–50% Matrigel (BD Biosciences, San Jose, California, USA) were injected into the left and right flanks of NOD/SCID/IL2Rγ−/− (NSG) mice, previously anesthetized intramuscularly with xylazine and tiletamine/zolazepam. Tumor growth was monitored over time by determining the volume of tumor masses. Mice with palpable tumor masses were randomized and treated for 3 weeks with doxycycline by oral administration (0.25 mg/ml for 6 days consecutively, then 4 days/week), CFZ i.v. (4 mg/kg biweekly), or the combination with the same dosing regimen used for the individual agents. Doxycycline was administrated in a 0.5% sucrose solution in light-proof bottles. CFZ was dissolved in 3% DMSO, 10% Captisol (CYDEX Pharmaceuticals Inc., Lenexa Kansas, USA), 10 mM sodium citrate pH 3.5, and administrated after doxycycline removal. The control group received the carriers alone at the same schedule as the combination group. Mice were euthanized in a carbon dioxide chamber, after the tumor masses reached a volume of approximately 1500 mm3, or at early signs of distress. Tumor volume was calculated using the ellipsoid formula 4/3 × π × ½ × (length × width × depth). Animals were housed in the animal facility of the Molecular Biotechnology Center (Torino, Italy), in accordance with guidelines approved by the local Ethical Animal Committee. Experimental approval was obtained from the Italian Ministry of Health.

### Zebrafish embryo xenografts

Approximately 250 KMS-28 TTA cells stably expressing DsRed fluorescent protein were injected (2 nL) into the yolk sac of each embryo with a manual microinjector (Eppendorf, Germany). Embryo were maintained at 30 °C in standard embryo medium supplemented by 0.003% PTU, 1 g L^−1^ glucose, and 5 mmol L^−1^ l-glutamine. The efficiency of tumor xenografts was evaluated 24 h post injection (hpi) by fluorescence microscopy using ZEISS Axio Observer inverted microscope (10× magnification). Xenograft positive embryos were placed into 96-well plates (1 embryo per well) and divided randomly into four experimental groups: DMSO, 2.5 nM CFZ, 2 µM SP2509, or the combination. Tumor growth was evaluated 72 hpi by fluorescence microscopy. Tumor xenograft volume of control and drug-treated animals was estimated at 72 hpi by measuring the area of fluorescence on photomicrographs and normalized to the signal obtained at 24 hpi. Images were processed using ImageJ program. All procedures were authorized by the Ethical Committee of the University of Torino and the Italian Ministry of Health.

### Statistical analysis

Statistical analyses were performed with GraphPad Prism 5.01 (GraphPad Software Inc.). Statistical significance of differences observed (in both in vitro and in vivo experiments) was determined by Student t test or one-way ANOVA (following Tukey’s multiple comparison tests); differences were considered significant when P value was < 0.05 (*), < 0.01 (**), < 0.001 (***), or < 0.0001 (****).

## Results

### Loss-of-function screenings converge on KDM1A/LSD1 as synthetic lethal target to the proteasome inhibitor carfilzomib

**Fig. 1:**
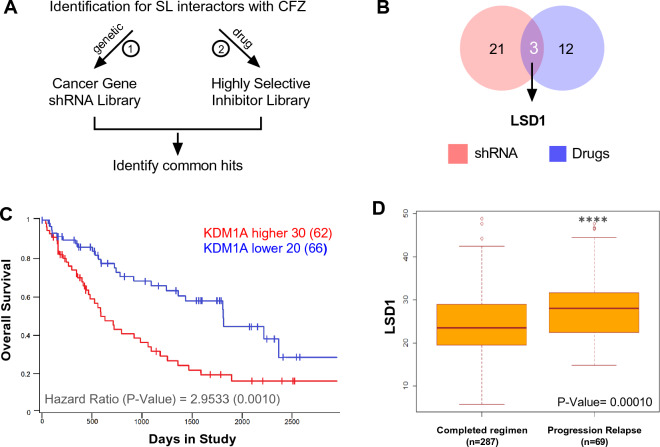
Loss-of-function screenings converge on KDM1A/LSD1 as synthetic lethal target to the proteasome inhibitor carfilzomib. **A** Schematic representation of the experimental strategy used in this study, showing the workflow of the loss-of-function screening to identify synthetic lethal targets for carfilzomib. SL, synthetic lethal. **B** Venn diagram of the top target hits found with shRNA and drug library screening indicating that KDM1A/LSD1 is a common hit from both screens. **C** Analysis of the MMRF CoMMpass dataset IA18 showed a significant correlation between LSD1 expression (RNAseq, TPM) and overall survival in 128 MM patients (P = 0.001). Sub-populations with low (blue line) or high (red line) LSD1 expression were defined using one standard deviation from the mean expression. **D** Box plots of gene expression levels in 287 MM cases that completed a regimen compared to 69 MM patients who experienced disease progression/relapse after bortezomib treatment (CoMMpass dataset). Differential expression was tested by the Wilcoxon rank-sum test with continuity correction (P = 0.0001018)

To identify druggable targets that synergize with PIs, we conducted two loss-of-function screenings by treating MM cells with sublethal concentrations of CFZ (Fig. 1A). First, we used a shRNA library, targeting 152 cancer driver genes, in the PI-resistant (PIR) multiple myeloma cell lines KMM-1^PIR^ and U266^PIR^ (Additional file 1: Fig. S1A), whose IC_50_ was previously calculated compared to naïve cell lines (Additional file 1: Table S1) [21]. We identified twenty-four hits, ten of which were shared between the two cell lines (Additional file 1: Fig. S1A). Second, we conducted a parallel drug screening using a library of 320 small-molecule inhibitors covering 123 key signaling targets (Additional file 1: Fig. S1B). U266^PIR^ cells were exposed to drugs library at 4 concentrations (10 μM, 1 μM, 100 nM, and 10 nM) in the presence or absence of a sublethal concentration of CFZ and analyzed after 72 h to calculate the Excess over Bliss (EOB) score. An arbitrary cut-off of EOB > 0.2 was used to define the top 15 synergistic candidates. KDM1A/LSD1, AKT, and EZH1/EZH2 emerged as overlapping hits in both screenings (Fig. 1B and Additional file 1: Fig. S1C, D). Notably, EZH1/EZH2 and the PI3K/Akt/mTOR pathway have been previously described to synergize with PIs in MM, thus enforcing our screening results [27, 28]. We then focused on the Lysine-Specific Histone Demethylase 1 (also known as KDM1A) for further investigations.

### LSD1 expression is increased in bortezomib-resistant MM patients and correlates to worse survival

LSD1 is overexpressed in numerous hematological and solid tumors, such as acute myeloid leukemia (AML), prostate, bladder, lymphoid neoplasm, and breast, among others [29]. To investigate the clinical and biological relevance of LSD1 expression in MM context, we took advantage of a large cohort of MM patients enrolled in the Multiple Myeloma Research Foundation CoMMpass study (https://research.themmrf.org/). Based on RNA sequencing data, we stratified patient’s population in LSD1-high and LSD1-low expression, and we found that high LSD1 expression correlates with worse overall survival (P = 0,001) and progression-free survival (P = 0.0006) (Fig. 1C and Additional file 1: Fig. S2A). Notably, LSD1 expression was significantly higher (P = 0.0001) in patients who experienced disease progression/relapse after bortezomib treatment compared to patients who completed the regimen (Fig. 1D), suggesting a potential role of LSD1 in drug resistance.

### LSD1 is a promising therapeutic target to combine with carfilzomib

**Fig. 2:**
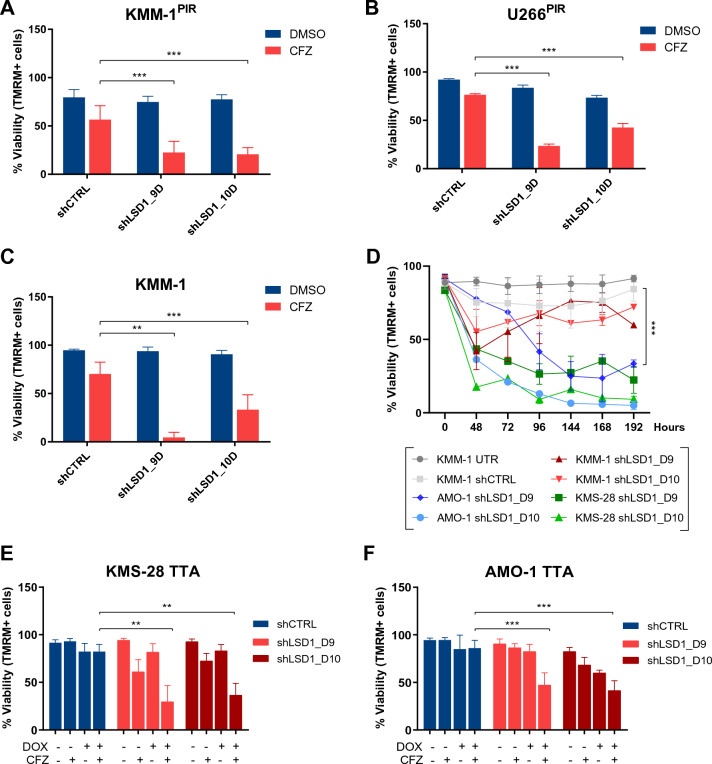
LSD1 silencing enhances sensitivity to CFZ in PI-resistant and PI-sensitive MM cell lines. **A** KMM-1^PIR^ and **B** U266^PIR^ cell lines were transduced with control shRNA (shCTRL) or shRNA targeting LSD1 (shLSD1_D9, shLSD1_D10) and treated with CFZ (20 nM for KMM-1^PIR^ and 10 nM forU266^PIR^) or DMSO. Cell viability was measured by TMRM staining-flow cytometry 72 hpt. **C** KMM-1 cell line was transduced with shCTRL or with indicated shLSD1 and treated with CFZ (2.5 nM). Cell viability was measured by TMRM staining-flow cytometry 72 hpt. **D** KMM-1, AMO-1, and KMS-28 cell lines were transduced with shCTRL or with indicated shLSD1 following puromycin selection (1.5 µg/ml). Cell viability was measured by TMRM staining-flow cytometry over time. **E** Inducible KMS-28 TTA and **F** inducible AMO-1 TTA cells were transduced with shCTRL or with reported shLSD1 and treated or not with DOX (1 µg/ml) for 120 h and then 72 h with CFZ (2.5 nM for KMS-28 and 1.25 nM for AMO-1). Cell viability was measured by TMRM staining-flow cytometry 72 hpt. Data are the means ± standard deviation (s.d.) of at least three independent experiments. Asterisks denote statistical significance (**P < 0.01; ***P < 0.001). PIR: proteasome inhibitors resistant; hpt: hours post-treatment; TMRM: tetramethylrhodamine; DOX: doxycycline. **E** Inducible KMS-28 TTA and **F** inducible AMO-1 TTA were transduced with shCTRL or with reported shLSD1 and treated or not with DOX (1 µg/ml) for 120 h and then 72 h with CFZ (2.5 nM for KMS-28 and 1.25 nM for AMO-1). Cell viability was measured by TMRM staining-flow cytometry 72 hpt

To validate screening results, two shRNAs (D9 and D10) directed against human LSD1 were individually transduced in PI-resistant MM cells. LSD1 silencing was confirmed by RT-qPCR and immunoblotting analyses (Additional file 1: Fig. S3A–C). While LSD1 knock-down did not affect viability in KMM-1^PIR^ and U266^PIR^, its combination with CFZ treatment significantly increased cell death (Fig. 2A, B). These findings prompted us to investigate whether LSD1 silencing could synergize with CFZ also in PI-sensitive cell lines. We first observed that LSD1 knockdown considerably enhanced sensitivity to CFZ in the parental KMM-1 cell line (Fig. 2C). However, LSD1 depletion was sufficient to affect cell viability in KMS-28 and AMO-1 cells (Fig. 2D). Thus, to better analyze the functional consequences of LSD1 silencing, we genetically modified KMS-28 and AMO-1 cells to express LSD1-shRNAs (D9 and D10) conditionally under the control of the doxycycline (DOX)-regulated transcriptional repressor tTR-KRAB (TTA) [30, 31]. We confirmed the progressive loss of LSD1 protein expression upon DOX treatment (Additional file 1: Fig. S3D–I). Remarkably, inducible LSD1 knockdown increased CFZ sensitivity of KMS-28 and AMO-1 cells (Fig. 2E, F). These results demonstrate that LSD1 is a feasible therapeutic target to combine with CFZ in both PI-resistant and sensitive MM cells.

### Pharmacological inhibition of LSD1 enhances sensitivity to carfilzomib in B-cell malignancies

**Fig. 3:**
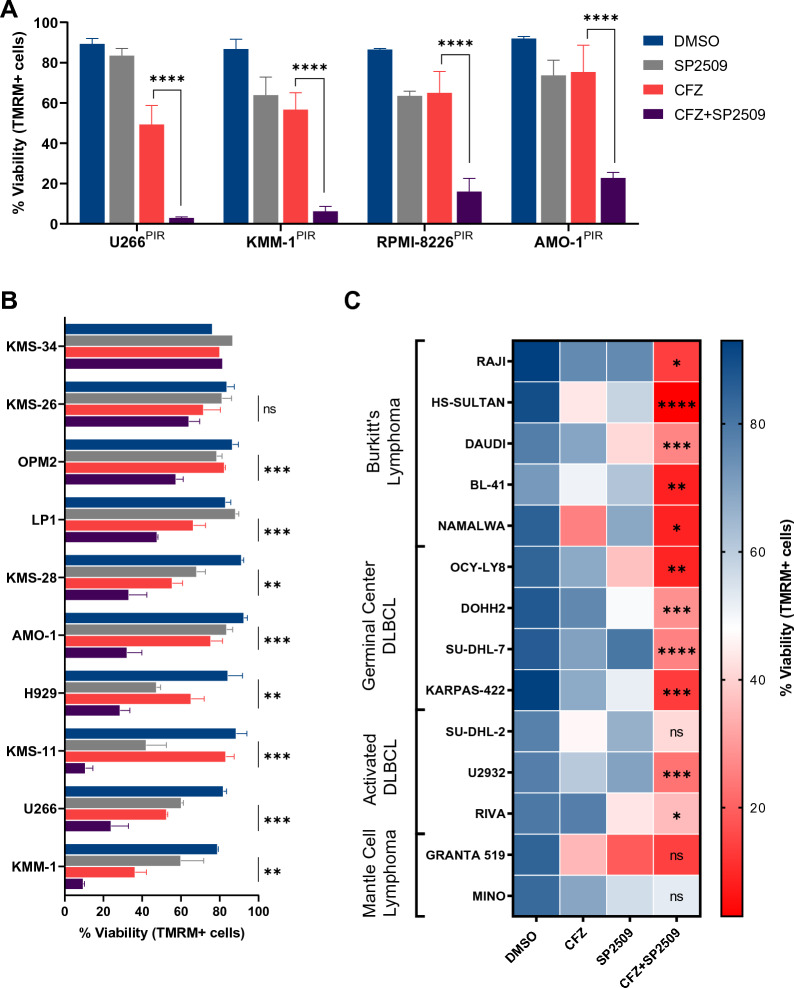
Pharmacological inhibition of LSD1 enhances sensitivity to CFZ in B-cell malignancies. **A** U266^PIR^ (20 nM CFZ; 0.5 µM SP2509), KMM-1^PIR^ (10 nM CFZ; 0.5 µM SP2509), RPMI-8226^PIR^ (60 nM CFZ; 1.5 µM SP2509), and AMO-1^PIR^ (20 nM CFZ; 0.5 µM SP2509) cells were treated with SP2509, CFZ or the combination. Cell viability was measured by TMRM staining-flow cytometry 48-, 120-, 72-, and 96- hpt, respectively. **B** MM cell lines were treated with CFZ (1.25 nM for AMO-1, OPM2, LP1, U266; 2.5 nM for KMS-28, KMM-1; 5 nM for KMS-11, KMS-34, KMS-26, NCI-H929), SP2509 (0.1 µM for KMS-11, KMS-26, KMS-34; 0.25 µM for KMS-28; 0.5 µM for AMO-1, H929, U266, KMM-1, OPM2, LP1), or the combination. Cell viability was measured by TMRM staining-flow cytometry spanning a range between 24 and 120 hpt. **C** Sensitivity heatmap to DMSO, CFZ, SP2509, or the combination in a panel of B-cell lymphoma cell lines. Cells were treated with CFZ (1.25 nM for BL-41; 2 nM for U2932; 2.5 nM for Mino, SU-DHL-2, Daudi, Namalwa, Riva, Granta519; 3.75 nM for Karpas-422; 5 nM for Raji, HS-Sultan, DOHH-2, SU-DHL-7; 7.5 nM for OCI-Ly8), SP2509 (0.25 µM for Namalwa; 0.5 µM for Raji, HS-Sultan, Daudi, OCI-Ly8, Granta-519; 0.75 µM for Mino, Riva, Karpas-422; 1 µM for DOHH-2, BL-41, Su-DHL-7), or the combination. Cell viability was measured by TMRM staining-flow cytometry at 72 or 96 hpt. Heatmap was generated using RStudio and ggplot2 package. Data are the means ± s.d. of at least three independent experiments. Asterisks denote statistical significance (*P < .05; **P < .01; ***P < .001; ****P < .0001; ns > .05). hpt: hours post-treatment

To investigate whether pharmacological inhibition of LSD1 could reproduce the synthetic lethal effect observed with genetic knockdown, we combined CFZ treatment with the reversible and non-competitive LSD1 inhibitor SP2509, which emerged as a top candidate in our drug library screen (Additional file 1: Fig. S1D). We found that SP2509 synergistically increased cell death when used in combination with CFZ in a panel of four PI-resistant MM cell lines (Fig. 3A, Additional file 1: Table S1) and in eight out of ten PI-sensitive cell lines (Fig. 3B), confirming that the synergy between LSD1 and proteasome inhibition is not limited to resistant cells. To further expand the clinical relevance of our findings, we evaluated the efficacy of SP2509/CFZ combination in a panel of B-cell non-Hodgkin lymphoma models such as diffuse large B-cell lymphoma (DLBCL), Burkitt’s lymphoma, and mantle cell lymphoma (MCL) cell lines. Cell viability analysis revealed that eleven out of fourteen cell lines displayed enhanced sensitivity to the combinatorial treatment compared to either agent alone (Fig. 3C). Collectively, these findings suggest that combining LSD1 and proteasome inhibition could represent a promising therapeutic approach for the treatment of MM and other B-cell hematological malignancies.

### LSD1 specifically mediates CFZ sensitivity in multiple myeloma

**Fig. 4:**
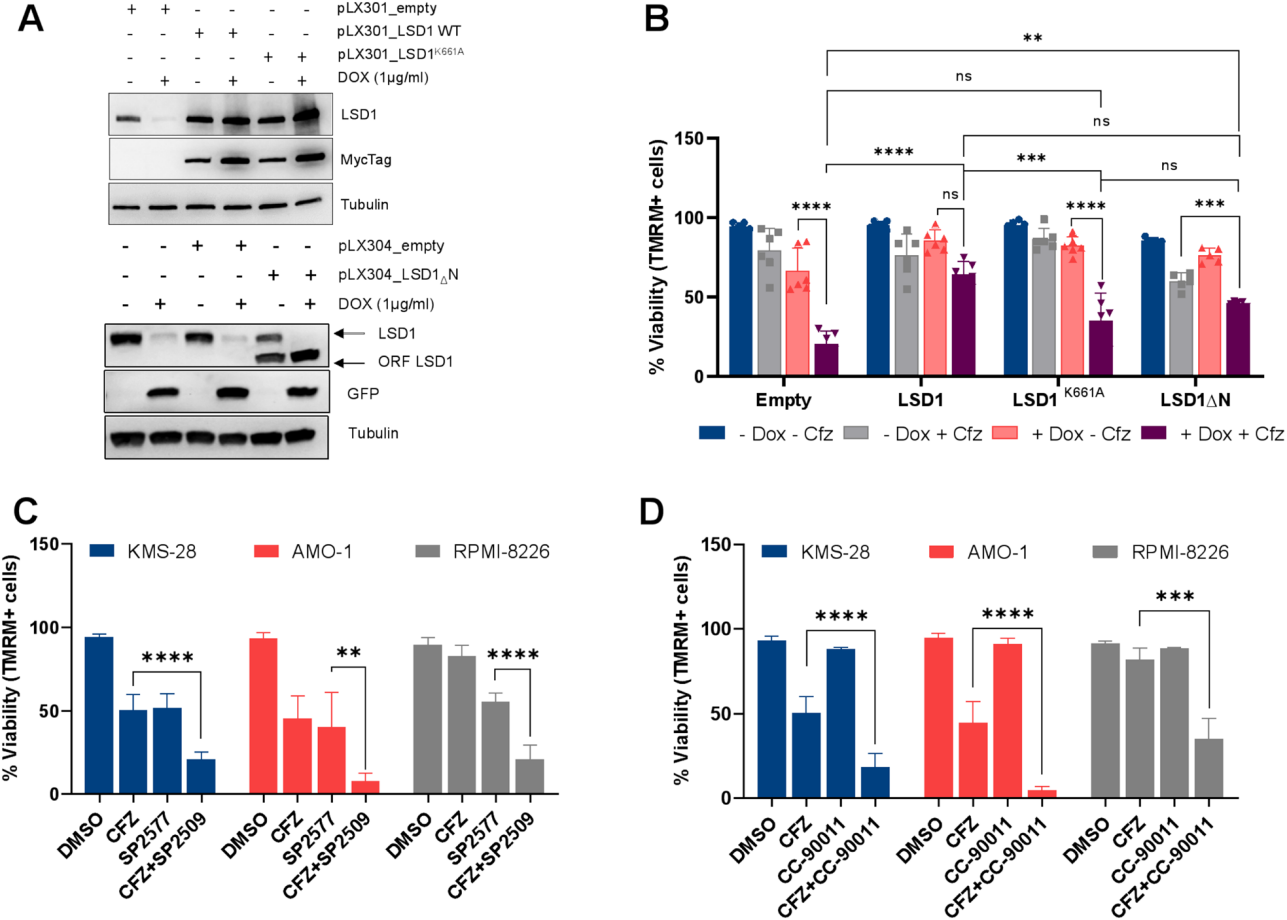
LSD1 specifically mediates CFZ sensitivity. **A** Inducible KMS-28 TTA_shLSD1-D6 cells were transduced with shRNA-resistant and Myc_tagged LSD1 WT (pLX301_LSD1 WT), catalytically inactive LSD1^K661A^ (pLX301_LSD1^K661A^), N-terminal lacking LSD1 (pLX304_LSD1_Δ_N) or empty vector (pLX301_empty, pLX304_empty). Cells were treated or not with DOX (1 µg/ml) and pellets collected after 5 days. LSD1, MycTag and GFP expression were analyzed by western blot. α-tubulin was used for protein loading normalization. **B** Inducible KMS-28 TTA_shLSD1-D6 cells were pre-treated with DOX (1 µg/ml) for 5 days, then with 2.5 nM CFZ. Cell viability was measured by TMRM staining-flow cytometry 120 h post-CFZ treatment. **C**, **D** Indicated cells were treated with SP2577 (2 µM for AMO-1 and RPMI-8226; 4 µM for KMS-28), CC-90011 (20 µM), CFZ (2.5 nM for KMS-28 and RPMI-8226; 5 nM for AMO-1) or the combinations. Cell viability was measured by TMRM staining-flow cytometry 72 hpt. Data are the means ± s.d. of at least three independent experiments. (**P < .01; ***P < .001; ****P < .0001; ns > .05)

To confirm the specificity of proteasome/LSD1 synthetic lethal interaction and exclude shRNA off-target effects, we transduced KMS-28 cells expressing doxycycline-inducible shLSD1 with an shRNA-resistant myc-tagged wild-type LSD1, its catalytically inactive K661A counterpart [32] or an N-terminal lacking form (Additional file 1: Fig. S3D–F). We first confirmed the modulation of LSD1 expression upon doxycycline treatment (Fig. 4A). Then, we observed that the ectopic expression of LSD1-WT fully rescued the cytotoxicity induced by LSD1 silencing in combination with CFZ, confirming that LSD1 activity modulates CFZ sensitivity (Fig. 4B). Conversely, forced expression of the LSD1^K661A^ or the N-terminal lacking mutants only partially rescued cell viability, suggesting that LSD1 full length is required to sustain the survival of CFZ-treated MM cells (Fig. 4B). The synergy between CFZ and LSD1 inhibition was further confirmed using SP2577 (seclidemstat), a SP2509 analog in clinical trial for advanced solid tumors (NCT03895684) and relapsed/refractory Ewing sarcomas (NCT03600649) [33] (Fig. 4C). Additionally, the reversible and potent LSD1 inhibitor, CC-90011 (pulrodemstat), displayed synergistic activity combined with CFZ in three PI-sensitive cell lines (Fig. 4D), while two irreversible and enzymatic LSD1 inhibitors, GSK2879552 and GSL-LSD1, did not induce any cytotoxic effect under any conditions (Additional file 1: Fig. S4A-B). We confirmed effectiveness of the latter drugs, treating AML cell line MOLM-13, known to be sensitive to LSD1 inhibition, with SP2509, GSK2879552 and GSK-LSD1. All treatments induced G_0_-G_1_ cell cycle arrest (Additional file 1: Fig. S4C) and cell differentiation, as demonstrated by the increase expression of surface CD11b protein (data not shown). Interestingly, MM cell lines treated with SP2509 showed cell cycle arrest in S and G_2_-M phases, suggesting its distinct and cell-context dependent mechanism of action (Additional file 1: Fig. S4D). Finally, we combined LSD1 inhibitor SP2509, with the FDA-approved proteasome inhibitors BTZ and ixazomib (IXZ) and observed synergistic activity in PI-sensitive and -resistant cell lines (Additional file 1: Fig. S4E, F). Taken together, these results confirmed that the synergy observed with CFZ is specifically related to LSD1 activities.

### CFZ/SP2509 treatment affects cell cycle regulation and induces apoptosis

**Fig. 5:**
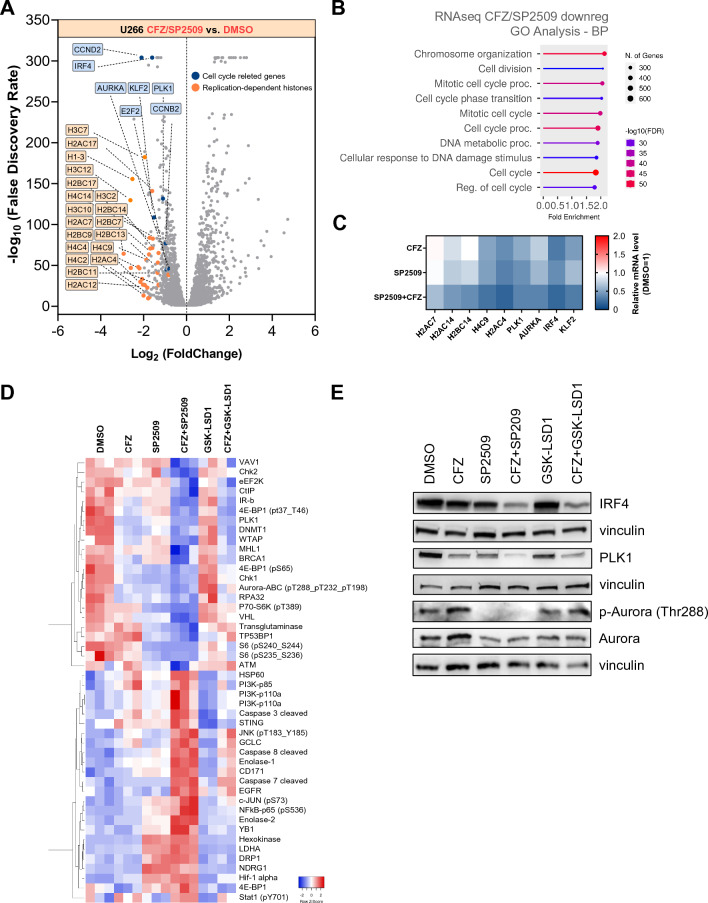
SP2509/CFZ treatment affects cell cycle regulation and apoptosis. **A** Volcano plot showing differentially expressed genes (DEGs) with |Log2FoldChange|> 0.5 between CFZ/SP2509 treatment and DMSO control. Analysis was performed 24 h post-treatment. Blue dots represent significantly downregulated genes related to cell cycle processes, orange dots represent significantly downregulated genes belonging to the replication-dependent histones family. **B** Dot plot graph of enriched Gene Ontology (GO) terms from genes in **A**. The nine GO processes with the largest gene ratios are plotted in order of fold enrichment. The size of the dots represents the number of genes in the significant DEG list associated with the GO term, and the color of the dots represents the P-adjusted values. **C** Representative heatmap of RT-qPCR validation performed in the U266 cell line treated with CFZ (2.5 nM), SP2509 (1 µM), or the combination for 48 h. HUPO was used as a housekeeping gene. DMSO-treated cells were used to normalize mRNA expression levels equal to 1. **D** Heatmap showing non-supervised hierarchical clustering of normalized reverse phase protein array (RPPA) intensities using the average-linkage method and Pearson distance. U266 cells were treated with DMSO (n = 3), CFZ (2.5 nM, n = 3), SP2509 (1 µM, n = 3), CFZ/SP2509 (n = 3), GSK-LSD1 (1 µM, n = 2), or CFZ/GSK-LSD1 (n = 2). Pellets were collected 48 hpt. **E** Western blot analysis of U266 cells treated with the indicated drugs. Pellets were collected 48 h post-treatment. Vinculin was used for protein loading normalization. DEG: differential expressed genes, GO: gene ontology; RPPA: reverse phase protein array

To investigate the molecular mechanisms underlying the observed synergies, we conducted RNA-sequencing and Reverse-Phase Protein Arrays (RPPA) experiments in U266 cells treated with either DMSO, CFZ, SP2509, GSK-LSD1 or the combinations. Analyses were performed 24 h post-treatment, when cell viability levels were comparable (Additional file 1: Fig. S5A). We first focused on genes deregulated by SP2509 alone or in combination with CFZ. The combination treatment led to a higher number of differentially expressed genes (DEGs) compared to single-agent treatments, likely due to CFZ’s pleiotropic effects (Additional file 1: Fig. S5B). CFZ/SP2509 combination significantly downregulated genes associated with cell cycle modulators, including PLK1, AURKA, CCND2, IRF4, KLF2, and a large cluster of replication-dependent histones (Fig. 5A). Gene ontology (GO) pathway analysis revealed an enrichment of pathways related to cell cycle control and chromosome organization (Fig. 5B). Independent RT-qPCR confirmed a significant downregulation of most of the genes identified by RNA-sequencing (Fig. 5C). Concordantly, RPPA analysis showed downregulation of cell cycle regulation and progression proteins, as well as deregulation of apoptotic proteins (Fig. 5D). Proteomic GO pathway analysis of the combination treatment confirmed an enrichment of pathways involved in G_1_-S phase transition and mitotic regulation (Additional file 1: Fig. S6A), suggesting that LSD1 and proteasome inhibition might act in concert to transcriptionally regulate protein expression. Indeed, we observed a grater accumulation of both mono- and di-methylation of histone 3 Lys4 (H3K4) and histone 3 Lys9 (H3K9) in the combination treatment compared to single agents (Additional file 1: Fig. S5C). Finally, we confirmed increased deregulation of proteins involved in cell cycle and apoptosis upon CFZ/SP2509 combination treatment, including downregulation of cyclin B, cyclin E, BCL2 and BCL-XL, upregulation of p21, cleaved caspases 3, 7, and 9 (Additional file 1: Fig. S5D, E). Interestingly, western blot analysis highlighted complete abrogation of p-Aurora kinase A (AURKA) and downregulation of IRF4 and PLK1 in CFZ/SP2509 compared to CFZ/GSK-LSD1 (Fig. 5F). Altogether, our data demonstrate that LSD1 and proteasome inhibition predominantly affect cell cycle progression by altering both chromatin organization, G1-S entry and mitotic progression, highlighting a synergistic and specific activity between SP2509 and CFZ.

### LSD1 and proteasome inhibition effects rely on the activation of DNA damage pathway

**Fig. 6:**
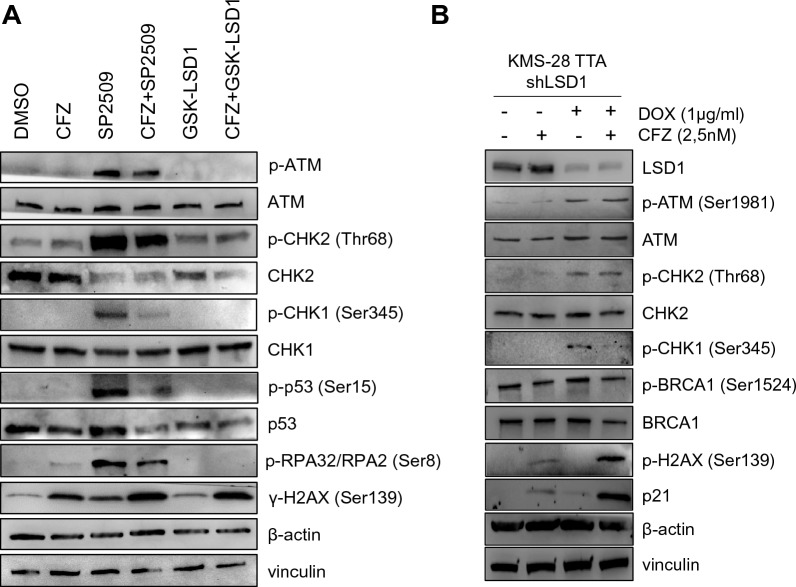
LSD1 and proteasome inhibition activate the DNA damage pathway. **A** U266 cells were treated with 2.5 nM CFZ, 1 µM SP2509, 1 µM GSK-LSD1, or the combinations. Cell pellets were collected at 48 h post-treatment: Expression of the indicated proteins was analyzed by Western blot. β-actin and vinculin were used as loading controls. **B** KMS28-TTA_shLSD1-D6 cells were pre-treated with DOX for 5 days and then treated with CFZ. Cell pellets were collected 72 h post-CFZ treatment, and protein expression was analyzed by Western blot. β-actin and vinculin were used as loading controls

To investigate the mechanisms through which CFZ/SP2509 combination impairs cell cycle progression and induces apoptosis, we compared CFZ/SP2509 and CFZ/GSK-LSD1 treatments (Fig. 5D; Additional file 1: Fig. S6B). RPPA heatmap analysis revealed that CFZ/SP2509 combination significantly deregulated DDR proteins (Fig. 5D), beside cell cycle and apoptosis. Accordingly, RNA-sequencing analysis of genes specifically deregulated by each LSD1 inhibitor in combination with CFZ (Additional file 1: Fig. S6B), confirmed the enrichment of DNA repair, mitotic cell cycle processes, and chromosome organization upon SP2509 treatment, whereas GSK-LSD1 mostly affected protein transport and localization to endoplasmic reticulum (ER), ribosome biogenesis, and nonsense-mediated decay process (Additional file 1: Fig. S6B). Western blot analysis in U266 cells confirmed that SP2509 alone or in combination with CFZ activated the DNA damage pathway by increasing the phosphorylation of ATM, CHK1, CHK2, p53, and H2A.X proteins (Fig. 6A). On the contrary, GSK-LSD1 treatment did not produce the same effects. To confirm the specificity of SP2509 in mediating DDR activation through LSD1 inhibition and exclude off-target effects, we treated KMS-28 TTA_shLSD1 cells with doxycycline to conditionally silence LSD1 in the presence or absence of CFZ treatment. We observed increased phosphorylation of most of DNA damage proteins and upregulation of p21 in cells treated with doxycycline and CFZ compared to single treatments (Fig. 6B). In summary, these results demonstrate that CFZ/SP2509 combination induces cell death by regulating the DNA damage pathway.

### LSD1 and proteasome inhibition have anti-MM activity in vivo

**Fig. 7:**
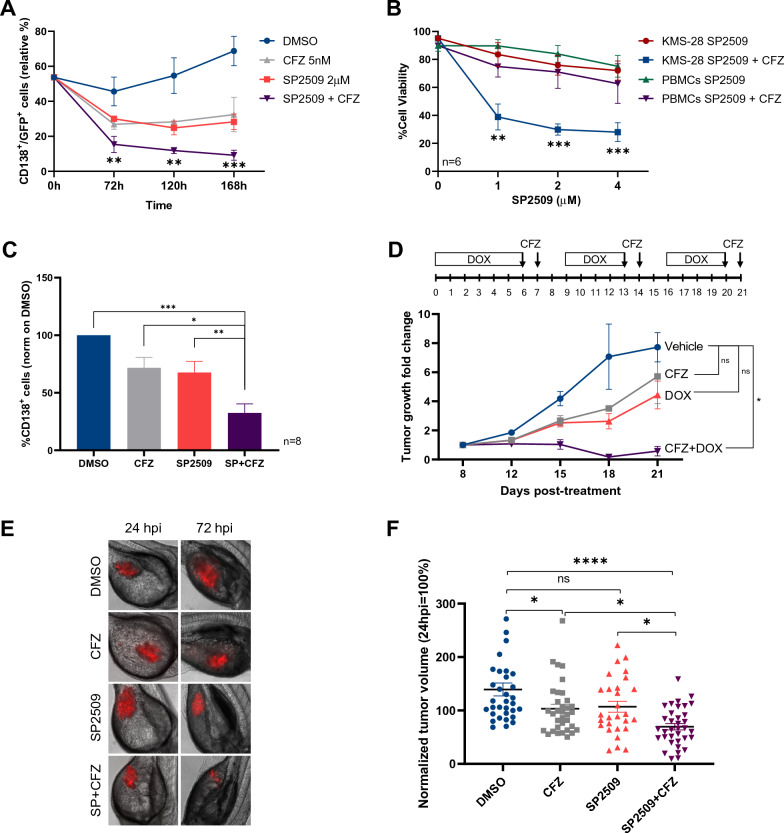
LSD1 and proteasome inhibition have anti-multiple myeloma activity in vivo. **A** U266 cell lines were cultured on a layer of GFP^+^ HS-5 stromal cells and treated with the indicated concentrations of SP2509, CFZ, or the combination. Percentage of MM cells was measured overtime by FACS analysis after anti-CD138-APC staining and the ratio with GFP + cells was calculated. Data are the means ± s.d. of four independent experiments **B** KMS-28 and PBMCs from six healthy donors were treated or not with 2.5 nM of CFZ and increasing concentration of SP2509, as reported. Cell viability was estimated by FACS analysis with Annexin V-PI staining, 72 hpt. Data are the means ± s.d. of 6 independent experiments. **C** Buffy coats derived from bone marrow aspirates of MM patients were treated with CFZ (2.5 nM) in combination or not with SP2509 (4 µM). Cell viability was estimated by flow cytometry measuring PI and CD138^+^ cells 72 hpt. Histograms represent the percentage of viable cells normalized vs DMSO samples. Data are the means ± s.e.m. of 8 independent MM patients. **D** Growth rate fold change (normalized on tumor volume measured 8 days post-treatment) of KMS-28-TTA_shLSD1 cells injected subcutaneously into the flanks of NSG mice. When tumor masses became palpable, mice were randomized for treatment with vehicle (n = 22), 4 mg/kg CFZ (n = 19), 0.25 mg/ml DOXY (n = 11), or a combination of both compounds (n = 7) over 3 weeks. The timeline above shows the schedule of treatment followed for in vivo treatments. **E** Fluorescent microscopy images (×10 magnification) of DsRed^+^KMS-28 TTA xenografts at 24 hpi (left) and 72 hpi (right) into the yolk of zebrafish embryos treated with indicated compounds. **F** Dot-plot shows the trend in tumor burden at 72 hpi, normalized to tumor area at 24 hpi (DMSO = 24 embryos; CFZ = 24 embryos; SP2509 = 24 embryos; SP2509/CFZ = 30 embryos). (**P < 0.01; ***P < 0.001; ****P < 0.0001). PBMC, Peripheral blood mononuclear cell; s.e.m.: standard error of the mean; hpi: hours post-injection

It is well established that bone marrow (BM) microenvironment provides nutrients and grow factors that sustain MM cell proliferation and can affect therapy resistance [34]. To evaluate the efficacy of SP2509/CFZ therapy in the presence of BM milieu, we co-cultured U266 MM cells with HS-5 stromal cells. Long-term experiments confirmed that CFZ/SP2509 treatment induced greater cell death in co-cultured U266 cells compared to single agents and untreated cells (Fig. 7A). Importantly, no negative effects were observed against stromal HS-5 cell line (Additional file 1: Fig. S7A). To further evaluate the therapeutic potential of combo therapy, we treated ex vivo cultures of CD138^+^ plasma cells from nine newly diagnosed MM patients (Table 1) and PBMCs from six healthy donors with CFZ/SP2509 or single agents. While the combination did not affect PBMCs viability (Fig. 7B), it significantly enhanced cell death of CD138+ MM primary cells, confirming the effectiveness of the combinatorial therapy even at the lowest SP2509 concentration (Fig. 7B, C). To provide in-vivo proof of principle that LSD1 inhibition could increase the therapeutic efficacy of PIs in MM, we exploited inducible shLSD1-expressing cells (Fig. 2E) [21]. Briefly, we subcutaneously injected KMS-28 TTA_shLSD1 cells into the flanks of NOD/SCID/IL2Rγ−/− (NSG) mice and when tumors became palpable, we treated them with doxycycline, CFZ, or control diluents. While single treatments had a moderate effect on tumor growth, the combination of LSD1 silencing and CFZ led to significant growth reduction (Fig. 7D), with no toxic effects toward treated mice (Additional file 1: Fig. S7B). To validate drug effectiveness, we used a zebrafish MM xenograft model. DsRed-positive KMS-28 TTA cells were injected into the yolk sac of 48 h post-fertilization (hpf) embryos. Xenotransplanted embryos were treated with DMSO, CFZ, SP2509 or the combination 24 h post injection (hpi) and visualized 72 hpi for tumor cell growth (Fig. 7E). CFZ/SP2509 treatment inhibited tumor growth of approximately 53% compared to control, while CFZ and SP2509 single treatments had only a moderate effect (29% and 23%, respectively). Overall, these results demonstrate that CFZ/SP2509 combination is a feasible therapeutic option to treat MM patients, with no evident side effects toward normal cells.

**Table 1:** Clinical and cytogenetic data of MM patients

N. Don	1	2	3	4	5	6	7	8	9
YOB	1966	1958	1954	1944	1961	1961	1962	1951	1949
% CD138	17.5	28.0	16.0	23.0	32.9	19.8	14.8	25.7	11.0
Gender	Male	Male	Male	Male	Female	Male	Male	Female	Male
SMM vs NDMM vs RRMM	NDMM	NDMM	SMM	NDMM	RRMM	NDMM	NDMM	NDMM	RRMM
Drug exposure	N/Ap	N/Ap	N/Ap	N/Ap	BTZ, THA, MEL, LEN	N/Ap	N/Ap	N/Ap	BTZ, THA, MEL, CFZ, LEN
Drug refractoriness	N/Ap	N/Ap	N/Ap	N/Ap	LEN	N/Ap	N/Ap	N/Ap	BTZ, THA
N. of prior lines	0	0	0	0	1	0	0	0	2
Disease isotype	IgG k	IgG l	IgA l	FLC k	FLC k	IgG l	IgG k	IgG k	IgG k
ISS at diagnosis	2	1	NP	N/A	2	1	3	1	2
Del(17p)	Neg	Neg	Neg	Neg	Pos	Neg	Neg	Pos	Pos
T(4;14)	Neg	Neg	Neg	Neg	Neg	Pos	Neg	Neg	N/A
T(14;16)	Neg	Neg	Neg	Neg	Neg	Neg	Neg	Neg	N/A
Gain/Amp(1q)	Neg	Neg	Neg	N/A	Pos	Pos	Neg	Pos	Neg
Del(1p)	Neg	Neg	Neg	N/A	Neg	Neg	Neg	Neg	Neg
T(11;14)	Neg	Neg	Pos	N/A	Pos	Neg	Neg	N/A	N/A
Del(13q)	Neg	Neg	Neg	N/A	Pos	Pos	Pos	N/A	Pos

YOB: Years of born; N/A: Not Available; N/Ap: Not Applicable; ISS: International Staging System; SMM: Smoldering MM; NDMM: New Diagnosed MM; RRMM: Relapse/refractory MM; BTZ: bortezomib; CFZ: carfilzomib; THA: Thalidomide; MEL: Melphalan; LEN: Lenalidomide

## Discussion

Our study employed comprehensive genetic and pharmacological screenings to identify LSD1 as a promising synthetic lethal target in combination with proteasome inhibitors (PIs) in multiple myeloma (MM) and other B-cell malignancies. LSD1 is an histone eraser involved in removing methyl groups fromH3K4me1/2, H3K9me1/2, as well as non-histone proteins, thereby exerting both transcriptional repression and activation and regulating protein stability [34, 35]. A correlation between LSD1 and tumor progression has been described in several malignancies and associated with unfavorable prognosis [36–40]. Due to its involvement in a plethora of cellular processes, LSD1 exhibits context-dependent effects. In numerous cancers, LSD1 is implicated in regulating key processes such as epithelial-to-mesenchymal transition (EMT), cancer invasion, and disease progression through its histone demethylating activity [42]. Additionally, LSD1 has been shown to regulate proliferation, angiogenesis, cell cycle arrest, and chromatin remodeling through the demethylation of non-histone proteins, including STAT3, HIF1α, E2F, and DNMT1 [43]. In the context of MM, the role of LSD1 remains poorly described and somewhat controversial. However, our study revealed increased LSD1 expression in MM patients who experienced disease relapse after bortezomib regimen therapy, suggesting a potential role for LSD1 in mediating drug resistance through epigenetic reprogramming. Notably, LSD1 physically interacts with Blimp-1 and other epigenetic factors during plasma blast differentiation, resulting in the repression of Blimp-1 target genes [41, 44]. LSD1 also modulates PB transcriptional networks by regulating chromatin accessibility and methylation level on several enhancers and PU.1, IRF4, and Blimp-1 binding sites, leading to the activation of key factors of MM pathogenesis, including c-MYC [45]. In our study, CFZ/SP2509 combination significantly reduced the expression of IRF4 and KLF2, genes known to be involved in B-cell lineage differentiation, further supporting the specific role of SP2509 in mediating LSD1 inhibition [46]. Additionally, LSD1 has been implicated in the regulation of p21WAF-1 in response to lenalidomide and pomalidomide treatment in a p53-dependent and -independent manner [46, 47]. In line with this, we observed increased p21 expression upon CFZ/SP2509 treatment. Moreover, our findings demonstrated that genetic and pharmacological LSD1 inhibition is synthetic lethal to proteasome inhibition in a broad panel of MM cell lines, as well as in primary MM cells regardless of disease stage, prior lines of therapy, or cytogenetic characteristics. This observation is particularly promising, as it suggests that our approach could be relevant for newly diagnosed multiple myeloma (NDMM) patients to prevent the emergence of a resistant phenotype [18]. The pivotal role of LSD1 in regulating B-cell homeostasis was further evidenced by its sensitivity in a panel of B-cell malignancies, including Burkitt's lymphoma and diffuse large B-cell lymphoma. These results underscore the clinical relevance of our study, suggesting that coupling epigenetic inhibitors like SP2509 with CFZ or other PIs could represent a promising therapeutic opportunity [48–50]. Notably, CFZ/SP2509 combination demonstrated a favorable cytotoxicity profile toward peripheral blood mononuclear cells and bone marrow–derived stromal cells. The clinical potential of LSD1-proteasome inhibition was further validated in MM xenograft models, resulting in tumor progression inhibition.

We also assessed the synergy of CFZ with various LSD1 inhibitors (LSD1i) [51–55] and observed that the allosteric inhibitors, SP2509 and SP2577, showed high synergistic effects with all FDA-approved PIs, whereas the enzymatic inhibitors GSK2879552 and GSK-LSD1 did not exhibit such effects in MM cells, despite inducing cell cycle arrest and differentiation in AML cells [56–58]. These results agree with our drug screening, that identified SP2509 as a top synergistic compounds and excluded enzymatic LSD1i, thus supporting the notion that LSD1's demethylase activity is dispensable in this context [56–58]. Also, the reversible and potent LSD1 inhibitor CC-90011 synergistically enhanced MM cell death, confirming the antitumoral potential of LSD1 and proteasome inhibition.

**Fig. 8:**
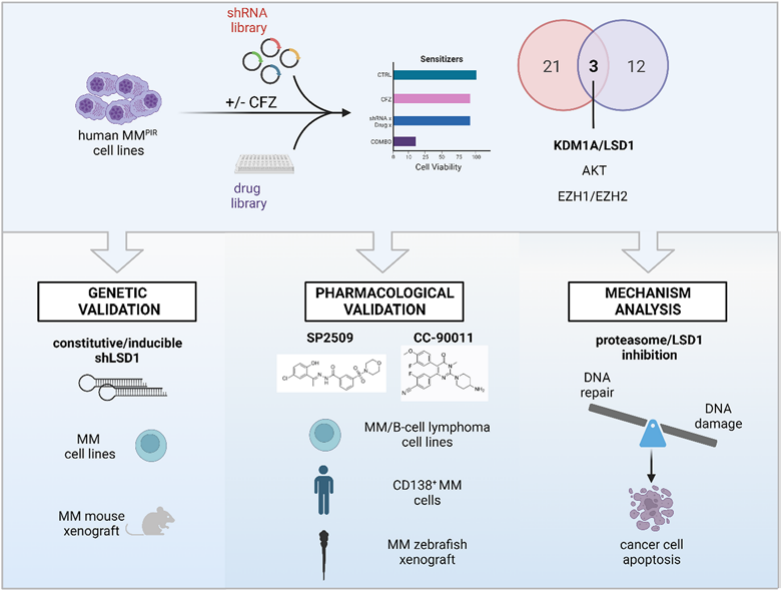
Genetic and pharmacologic inhibition of LSD1 is synthetic lethal with CFZ enhancing DNA damage in multiple myeloma and other B-cell neoplasms. Representative scheme indicating the study workflow and derived results

Mechanistically, we demonstrated that CFZ and SP2509 treatments lead to deregulation of chromosomal organization, cell cycle, and DNA damage checkpoints, ultimately activating the DNA damage pathway with phosphorylation of ATM, CHK1, CHK2, p53, and H2AX proteins. Interestingly, we observed that although both scaffolding and enzymatic LSD1 inhibitors induced yH2AX (Ser139) phosphorylation, suggesting DNA damage, carfilzomib further potentiated this effect, with SP2509 combination exhibiting additional changes. These results agree with rescue experiments showing that a full length LSD1 protein is necessary to modulate CFZ sensitivity, as catalytically inactive and N-terminal lacking forms of LSD1 failed to rescue cell viability. Our hypothesis is that CFZ treatment causes cell cycle alteration and DNA damage, while, LSD1 silencing or allosteric inhibition prevents its ability to interact and regulate DDR proteins not allowing the correct DNA repair and thus ultimately leading to cell apoptosis (Fig. 8). The link between proteasome inhibition and the DNA damage response (DDR) pathway has been established in various models, with proteasome inhibition impacting DDR through regulating nuclear foci formation, reducing homologous recombination (HR), and modulating repair protein expression or degradation [59–62]. While this suggests that CFZ mediates DNA damage through its on-target activity, specifically by inhibiting proteasomal degradation and disrupting protein turnover, further analyses are required to validate this mechanism in our specific model.

LSD1 is known to be recruited to DNA damage foci during the S-G_2_ phase, where it facilitates the interactions of RNF168, 53BP1 and BRCA1 to the DNA damage sites [63]. Interestingly, LSD1 binding to RNF168 relies on the N-terminal region of LSD1 protein, indicating that both demethylases-dependent and demethylases-independent activities may contribute to orchestrating the DDR response. Nevertheless, detailed signaling mechanisms for LSD1 competitive binding with DNA and other factors in MM remain to be determined. Emerging evidence suggests that LSD1 plays important roles in regulating the timing and efficiency of origin firing, chromosome segregation and mitosis [64, 65]. In this context, it is known that PLK1 phosphorylates LSD1 at Ser-126 promoting its release from chromatin during mitosis, thus balancing methylation levels during cell cycle [57, 66]. Moreover, LSD1 inhibition significantly affects G_2_-M DNA damage checkpoint regulation in solid tumors [67]. Here, we showed that CFZ/SP2509 combination abrogated PLK1 and AURKA expression, required for G_2_-M transition [67–69], with concomitant downregulation of cyclin B and upregulation of p21. However, whether this regulation is dependent on LSD1 demethylating activity at nucleosomes or proteins level requires further investigation. More extensive analyses are needed to correlate target gene expression levels with histone and DNA methylation profiles. Interestingly, our study, along with other recent findings, highlights the significance of epigenetic regulation in modulating drug resistance in myeloma. In particular, inhibition of euchromatic histone-lysine *N*-methyltransferase 2 (EHMT2) has been demonstrated as a valuable strategy to enhance PI sensitivity and overcome drug resistance in MM patients [70, 71]. This suggests that fine-tuning methylation/demethylation homeostasis is essential for MM cell survival.

## Conclusions

In summary, our study provides novel insights into the mechanisms underlying resistance to proteasome inhibitors in multiple myeloma and identifies LSD1 as a potential therapeutic target to enhance cell death in both resistant and non-resistant MM cells. Our findings support the development of new allosteric LSD1 inhibitors, as well as clinical trials combining them with PIs to overcome resistance and improve patient outcomes across a broad range of diseases, potentially including solid tumors.

## Supplementary Information

**Additional file 1. Additional Materials and Methods.** Cell culture conditions and reagents. MM patients and healthy donors samples. Virus production and in vitro transduction. LSD1 constructs and mutagenesis. Inducible shLSD1. shRNA screening. Library preparation and RNA-sequencing. Reverse Phase Protein Array. Purification of total RNA and Reverse Transcription-quantitative Polymerase Chain Reaction (RT-qPCR). Western Blotting. Analysis of apoptosis and cell cycle. ATPlite Assay. Zebrafish housing. **Additional References. Additional Figures and Legends. Figure S1.** Loss-of-function screenings performed in PI-resistant MM cell lines. **Figure S2.** CoMMpass database investigation. LSD1 expression is increased in bortezomib-resistant MM patients and correlates to worse survival. **Figure S3.** Analysis of LSD1 silencing using constitutive and inducible shLSD1. **Figure S4.** LSD1 inhibitors testing. **Figure S5.** SP2509/CFZ treatment is associated with antiproliferative and apoptotic programs. **Figure S6.** SP2509 but not GSK-LSD1 synergize with CFZ in MM cells. **Figure S7.** CFZ/SP2509 combinatorial treatment is not toxic to normal cells. **Additional Tables. Table S1.** IC50 of PIR-MM cells used in the present study. **Table S2.** shRNA sequences used in the present study. **Table S3.** Primer sequences used in the present study. **Table S4.** Antibodies used in the present study.

## Data Availability

The datasets generated and/or analyzed during the current study are available from the corresponding author on reasonable request.

## Abbreviations

MM: Multiple myeloma
PIs: Proteasome inhibitors
BTZ: Bortezomib
CFZ: Carfilzomib
IXZ: Ixazomib
PBMCs: Peripheral blood mononuclear cells
BMSCs: Bone marrow stromal cells
RNA-Seq: RNA-sequencing
RPPA: Reverse-Phase Protein Arrays
LSD1: Lysine-specific demethylase 1
IDH2: Isocitrate dehydrogenase 2
EHMT2: Euchromatic histone-lysine *N*-methyltransferase 2
DDR: DNA damage response
ER: Endoplasmic reticulum
UPR: Unfolded-protein response
PSMB5: Proteasome subunit Β type 5
EZH2: Enhancer of Zeste 2 Polycomb Repressive Complex 2 Subunit
KDM: Lysine Demethylase
HDACs: Histone Deacetylases
PIR: PI-resistant
EOB: Excess over Bliss
GR: Growth Rate
AML: Acute myeloid leukemia
DLBCL: Diffuse large B-cell lymphoma
MCL: Mantle cell lymphoma
DOX: Doxycycline
RT-qPCR: Reverse Transcription-quantitative Polymerase Chain Reaction
TTA: TTR-KRAB
DEGs: Differentially expressed genes
BM: Bone Marrow
NSG: NOD/SCID/IL2Rγ−/− mice
hpi: Hours post injection
hpf: Hours post-fertilization
NDMM: Newly diagnosed multiple myeloma
LSD1i: LSD1 inhibitors
