# Correction: Lysin (K)-specific demethylase 1 inhibition enhances proteasome inhibitor response and overcomes drug resistance in multiple myeloma

**DOI:** 10.1186/s40164-025-00675-y

**Published:** 2025-06-13

**Authors:** Cecilia Bandini, Elisabetta Mereu, Tina Paradzik, Maria Labrador, Monica Maccagno, Michela Cumerlato, Federico Oreglia, Lorenzo Prever, Veronica Manicardi, Elisa Taiana, Domenica Ronchetti, Mattia D’Agostino, Francesca Gay, Alessandra Larocca, Lenka Besse, Giorgio Roberto Merlo, Emilio Hirsch, Alessia Ciarrocchi, Giorgio Inghirami, Antonino Neri, Roberto Piva

**Affiliations:** 1 Department of Molecular Biotechnology and Health Sciences, University of Turin, Turin, Italy; 2 Department of Physical Chemistry, Rudjer Boskovic Insitute, Zagreb, Croatia; 3 Laboratory of Translational Research, Azienda USL-IRCCS Reggio Emilia, Reggio Emilia, Italy; 4 Hematology, Fondazione Cà Granda IRCCS Policlinico, Milan, Italy; 5 Department of Oncology and Hemato-Oncology, University of Milan, Milan, Italy; 6 Città Della Salute e della Scienza Hospital, Turin, Italy; 7 Experimental Oncology and Hematology, Department of Oncology and Hematology, St. Gallen Cantonal Hospital, St. Gallen, Switzerland; 8 Scientific Directorate, Azienda-USL IRCCS di Reggio Emilia, Reggio Emilia, Italy; 9 Department of Biology, Faculty of Medicine, Masaryk University, Brno, Czech Republic; 10 Department of Pathology and Laboratory Medicine, Weill Cornell Medicine, New York, NY USA

**Correction: Experimental Hematology & Oncology (2023) 12:71**

10.1186/s40164-023-00434-x

In this article [1] the legend for Fig. 2, panel C and D was inadvertently swapped; the figure should have appeared as shown below.

Original publication: **C** KMM-1, AMO-1, and KMS-28 cell lines were transduced with shCTRL or with indicated shLSD1 following puromycin selection (1.5 µg/ml). Cell viability was measured by TMRM staining-flow cytometry over time. **D** KMM-1 cell line was transduced with shCTRL or with indicated shLSD1 and treated with CFZ (2.5 nM). Cell viability was measured by TMRM staining-flow cytometry 72 hpt.

Corrected version: **C** KMM-1 cell line was transduced with shCTRL or with indicated shLSD1 and treated with CFZ (2.5 nM). Cell viability was measured by TMRM staining-flow cytometry 72 hpt. **D** KMM-1, AMO-1, and KMS-28 cell lines were transduced with shCTRL or with indicated shLSD1 following puromycin selection (1.5 µg/ml). Cell viability was measured by TMRM staining-flow cytometry over time.

The original article has been corrected.
