# Supplementary material for: Lysin (K)-specific demethylase 1 inhibition enhances proteasome inhibitor response and overcomes drug resistance in multiple myeloma
Source: Exp Hematol Oncol. 2023 Aug 10;12:71. doi: 10.1186/s40164-023-00434-x (PMC10413620; doi:10.1186/s40164-023-00434-x)
Supplement: Supplementary file 1 — Additional file 1. Additional Materials and Methods. Cell culture conditions and reagents. MM patients and healthy donors samples. Virus production and in vitro transduction. LSD1 constructs and mutagenesis. Inducible shLSD1. shRNA screening. Library preparation and RNA-sequencing. Reverse Phase Protein Array. Purification of total RNA and Reverse Transcription-quantitative Polymerase Chain Reaction (RT-qPCR). Western Blotting. Analysis of apoptosis and cell cycle. ATPlite Assay. Zebrafish housing. Additional References. Additional Figures and Legends. Figure S1. Loss-of-function screenings performed in PI-resistant MM cell lines. Figure S2. CoMMpass database investigation. LSD1 expression is increased in bortezomib-resistant MM patients and correlates to worse survival. Figure S3. Analysis of LSD1 silencing using constitutive and inducible shLSD1. Figure S4. LSD1 inhibitors testing. Figure S5. SP2509/CFZ treatment is associated with antiproliferative and apoptotic programs. Figure S6. SP2509 but not GSK-LSD1 synergize with CFZ in MM cells. Figure S7. CFZ/SP2509 combinatorial treatment is not toxic to normal cells. Additional Tables. Table S1. IC50 of PIR-MM cells used in the present study. Table S2. shRNA sequences used in the present study. Table S3. Primer sequences used in the present study. Table S4. Antibodies used in the present study. [file 40164_2023_434_MOESM1_ESM.docx]

**ADDITIONAL FILE 1**

**Lysin (K)-Specific Demethylase 1 Inhibition Enhances Proteasome Inhibitor Response and Overcomes Drug Resistance in Multiple Myeloma**

Cecilia Bandini^1^, Elisabetta Mereu^1^, Tina Paradzik^1,2^, Maria Labrador^1^, Monica Maccagno^1^, Michela Cumerlato^1^, Federico Oreglia^1^, Lorenzo Prever^1^, Veronica Manicardi^3^, Elisa Taiana^4,5^, Domenica Ronchetti^4,5^, Mattia D’Agostino^1,6^, Francesca Gay^1,6^, Alessandra Larocca^1,6^, Lenka Besse^7,8^, Giorgio Roberto Merlo^1^, Emilio Hirsch^1^, Alessia Ciarrocchi^3^, Giorgio Inghirami^9^, Antonino Neri^10^ and Roberto Piva^1,6^

^1^Department of Molecular Biotechnology and Health Sciences, University of Turin, Turin, Italy; ^2^Department of Physical Chemistry, Rudjer Boskovic Insitute, Zagreb, Croatia; ^3^Laboratory of Translational Research, Azienda USL-IRCCS Reggio Emilia, Reggio Emilia, Italy; ^4^Hematology, Fondazione Cà Granda IRCCS Policlinico, Milan, Italy; ^5^Department of Oncology and Hemato-oncology, University of Milan, Milan, Italy; ^6^Città Della Salute e della Scienza Hospital, Turin, Italy; ^7^Experimental Oncology and Hematology, Department of Oncology and Hematology, St. Gallen Cantonal Hospital, St. Gallen, Switzerland; ^8^Department of Biology, Faculty of Medicine, Masaryk University, Brno, Czech Republic; ^9^Department of Pathology and Laboratory Medicine, Weill Cornell Medicine, New York, NY, USA; ^10^Scientific Directorate, Azienda-USL IRCCS di Reggio Emilia, Reggio Emilia, Italy.

**Additional Materials and Methods**

Cell culture conditions and reagents. MM patients and healthy donors samples. Virus production and in vitro transduction. LSD1 constructs and mutagenesis. Inducible shLSD1. shRNA screening. Library preparation and RNA-sequencing. Reverse Phase Protein Array. Purification of total RNA and Reverse Transcription-quantitative Polymerase Chain Reaction (RT-qPCR). Western Blotting. Analysis of apoptosis and cell cycle. ATPlite Assay. Zebrafish housing.

**Additional References**

**Additional Figures and Legends**

**Figure S1.** Loss-of-function screenings performed in PI-resistant MM cell lines. **Figure S2.** CoMMpass database investigation. LSD1 expression is increased in bortezomib-resistant MM patients and correlates to worse survival. **Figure S3.** Analysis of LSD1 silencing using constitutive and inducible shLSD1. **Figure S4.** LSD1 inhibitors testing. **Figure S5.** SP2509/CFZ treatment is associated with antiproliferative and apoptotic programs. **Figure S6.** SP2509 but not GSK-LSD1 synergize with CFZ in MM cells. **Figure S7.** CFZ/SP2509 combinatorial treatment is not toxic to normal cells.

**Additional Tables**

**Table S1.** IC50 of PIR-MM cells used in the present study. **Table S2.** shRNA sequences used in the present study. **Table S3.** Primer sequences used in the present study. **Table S4.** Antibodies used in the present study.

**Additional Materials and Methods**

**Cell culture conditions and reagents**

Human multiple myeloma (MM) cell lines KMM-1, U266, KMM-1^PIR^, U266^PIR^, KMS-11, NCI-H929, LP-1, AMO-1, OPM-2, KMS-28, KMS-26, KMS-34, RPMI-8226; human Burkitt’s Lymphoma (BL) cell lines RAJI, HS-Sultan, Daudi, BL-41, Namalwa; human mantle cell lymphoma (MCL) cell lines Granta-519, Mino; human diffuse large B-cell lymphomas (DLBCL) SU-DHL-2, RIVA, U2932 (ABC-DLBCL), OCI-LY8, SU-DHL-7, DoHH2, KARPAS- 422 (GCB-DLBCL); human acute myeloid leukemia (AML) cell line MOLM-13 were obtained from DSMZ (German Collection of Microorganisms and Cell Cultures, Braunschweig, Germany), ATCC (American Type Culture Collection, Manassas, Virginia, USA), or generated in our lab and authenticated by DNA fingerprinting using GenePrint system (Promega, Madison, Wisconsin, USA). AMO-1^PIR^ and RPMI-8226^PIR^ were kindly provided by Dr. Lenka Besse (from Experimental Oncology and Hematology, Department of Oncology and Hematology, Kantonsspital St Gallen, St Gallen, Switzerland) [1,2]. Cell lines were maintained in RPMI 1640 medium (EuroClone, Pero, Italy), supplemented with 2 mM of L-glutamine, 100 U/mL of penicillin, 100 μg/mL of streptomycin (Gibco), 10-20% fetal bovine serum (FBS; Sigma-Aldrich, St. Louis, Missouri, USA), and growing at 37°C in a humidified atmosphere with 5% CO2. 293T cells obtained from DSMZ were cultured under standard conditions (37°C in humidified atmosphere, with 5% CO2) in DMEM supplemented with 10% FBS. Carfilzomib (PR-171), bortezomib (PS-341), ixazomib (MLN9708), SP2509, SP2577, CC-90011, GSK-LSD1 and GSK2879552 were obtained from Selleckchem (Munich, Germany) and from MedChemtronica (Stockholm, Sweden).

**MM patients and healthy donors samples**

Peripheral Blood Mononuclear Cells (PBMCs) from healthy donors and BM aspirates of MM patients were kindly provided by the local Blood Bank (Città Della Salute e della Scienza Hospital, Turin). PBMCs were collected from buffy coat by Ficoll-Hypaque density gradient separation, suspended in RPMI-10% FBS medium, and seeded (5x10^5^ cells/mL). BM cells were isolated by Ficoll-Hypaque gradient centrifugation, according to Milteny’s [protocol.](chrome-extension://efaidnbmnnnibpcajpcglclefindmkaj/https:/static.miltenyibiotec.com/asset/150655405641/document_k6fntaifc914b2p8io70utq50a/SP_MC_BM_density_gradient.pdf?content-disposition=inline)  CD138^+^ cells were detected by FACS analysis by using anti-CD138-APC (cat.no.130-117-395, Clone 44F9, Milteny). Samples containing more than 10% of CD138 myeloma cells were selected for further analysis, seeded (2,5x10^5^ cells/mL) in RPMI-10% FBS medium, and treated as described in the main manuscript.

**Virus production and in vitro transduction**

High titer lentiviral stocks were produced in 293T cells by co-transfecting the expression vector (in pLKO, pLX301 or pLVTHM backbones) and packaging vectors (pCMVdR8.74, VSV-G/pMD2.G) with the Effectene Transfection Reagent (Qiagen, Milan, Italy), according to the manufacturer's instructions. Supernatants were harvested over 36 to 60 hours, filtrated (0.22 μm pore), and used directly or concentrated by Lenti-X concentrator (ClonTech) according to the manufacturer's instructions, resuspended in cold phosphate-buffered saline (PBS), and then stored at -80°C. Aliquots of virus, plus 4-8 μg/mL polybrene, were used to infect KMM-1, KMM-1^PIR^, U266, U266^PIR^, KMS-28 and AMO-1 cells (1 x 10^5^/mL). Fresh medium was supplemented 2 hours after infection. Stable cell lines expressing indicated constructs were selected by treatment with 1 μg/mL puromycin (Sigma-Aldrich) for 24 or 48 hours.

**LSD1 constructs and mutagenesis**

LSD1 Myc-tag was amplified by PCR from pTRE2-LSD1 kindly provided by Prof. Tim C.P. Somervaille [3] (The University of Manchester, Manchester, United Kingdom) using Expand High Fidelity (Merk) and the following primers: LSD1_SalI_Fv: 5’-attGTCGACaccatgttatctgggaagaaggcggcag-3’, LSD1_XhoI_Rv: 5’- cacCTCGAGtcacagatcctcttctgagatgagtttttgttcacccgaaccCATGCTTGGGGACTGCTGTGCA-3’. The insert was digested with SalI and XhoI, purified with a QIAquick Gel Extraction Kit (Qiagen) and dephosphorylated. The insert was ligated into pENTR1A no ccdB vector using T4 ligase. The ligation mixture was transformed into Stabl3 competent cells. Lentiviral expression vector pLX301_LSD1 Myc-tag was generated by Gateway recombination (Gateway System, Invitrogen). Positive clones were identified by Sanger sequencing. To make the K661A mutant vector, the following primers were used in a site-direct mutagenesis reaction (QuikChange II Site-Directed Mutagenesis Kit, Agilent), following manufacturer’s instructions, using pLX301_LSD1 Myc-tag as a template: Fv: 5’-gggatttggcaaccttaacgcggtggtgttgtgttttgatc-3’, Rv: 5’-gatcaaaacacaacaccaccgcgttaaggttgccaaatccc-3’. N-terminal lacking LSD1 (pLX304_LSD1_Δ_N) was purchased from DNASU plasmid repository (HsCD00438895) (https://dnasu.org/DNASU/Home.do).

**Inducible shLSD1**

pLVTHM-GFP-shLSD1 vector was constructed by subcloning the U6 promoter–shLSD1-D6, shLSD1-D9, shLSD1-D10, and a shRNA control cassette into the EcoRI-ClaI sites of the pLVTHM vector [4], kindly provided by D. Trono (University of Geneva, Geneva, Switzerland). The shRNA sequences are reported in Additional Table S2. For conditional RNAi, KMS-28 cell line was transduced at high efficiency with pLV-DsRed-tTRKRAB plasmid (TTA), expanded, and used for transduction with pLVTHM-GFP-shLSD1 lentiviral particles. Next, cells were treated with doxycycline (1 μg/mL) for 12 hours, double GFP+DsRed+ cells were flow sorted (FACSAriaIII, BD Bioscience, Milan, Italy) and expanded. KMS-28 TTA_shLSD1 were checked for LSD1 silencing by doxycycline (DOX) treatment (1 μg/mL).

**shRNA screening**

shRNA screening was performed as previously described [5]. Briefly, a shRNA library targeting 152 cancer driver genes was assembled with 684 lentiviral shRNA (pLKO backbone) from The RNAi consortium (TRC - <https://www.broadinstitute.org/rnai-consortium/rnai-consortium-shrna-library>) (for the completed list of genes and sequences see ref.[5]). KMM-1^PIR^ cells were pre-seeded for 24 hours at a density of 20 000 cells/well, following infection with shRNA lentiviral supernatants at a MOI of 0.3 (day -3). After 24 hours the cells were selected with puromycin (2.5 μg/mL) (day -2). Percentage of transduction (T) of infected cells were calculated using Cell Titer Glo (Promega) luminescence assay detection, performed in duplicate two days after selection (day 0). At day 0 KMM-1^PIR^ cells were splitted and treated with 2.5 nM carfilzomib (CFZ) or with control diluent (DMSO) every 72 hours and Cell Titer Glo performed 3- and 7-days posttreatment. Luminescence values were used to calculate Cell Growth (CG), Growth Rate (GR), and Z-Score for each time point (for formulas see ref.[5]). We selected samples with Z-score lower than -0.75 (162 shRNAs) at day 3, and -0.8 (195 shRNA) at day 7. Within these groups, candidate genes were selected according to the following criteria: more than one shRNA sequence per gene determined growth inhibition in presence of CFZ; shRNA reduced target gene expression by at least 60%; positive hits were present both at day 3 and day 7 (19 genes), or within the top 5 at day 7 (5 genes). Top 24 scoring genes were validated in U266^PIR^ cell line using similar protocol for the primary screening. Correlation analysis between gene silencing and phenotype was used to define top three candidates.

**Library preparation and RNA-sequencing**

RNA-sequencing was performed as previously described [6]. Briefly, total RNA was extracted with Maxwell®RSC simplyRNA Cells (Promega). RNA-seq libraries were obtained from 100 ng of total RNA following Illumina Stranded TotalRNA Prep Ligation with Ribo-zero Plus protocol (Illumina). Sequencing was performed using Illumina NextSeq500 high-output cartridge (double-stranded, reads length 75bp-2×75 cycles). Sequencing quality was assessed using the FastQC v0.11.8 software (www.bioinformatics.babraham.ac.uk/projects/fastqc/). Raw sequences were aligned to the human reference transcriptome (GRCh38, Gencode release 35) using STAR [7] version 2.7 and gene abundances were estimated with RSEM algorithm (v1.3.1). Differential expression analysis was performed using Deseq2 R package [8], considering a False Discovery Rate (FDR) of 5% and excluding genes with low read counts.

**Reverse Phase Protein Array**

RPPA analyses were performed at the UT MD Anderson Cancer Center’s Functional Proteomics RPPA Core facility. U266 were seeded at 1 x 105 cells/ml in T75 cell culture flasks and treated with DMSO, 2,5 nM CFZ, 1 µM SP2509, or the combination. Cell viability was monitored over time up to 120 hours, and pellets collected at 24- and 48 hours post-treatment. Proteins were extracted using Lysis Buffer containing 1% Triton X‐100, 50mM HEPES, pH 7.4, 150mM NaCl, 1.5mM MgCl2, 1mM EGTA, 100mM NaF, 10mM Na pyrophosphate, 1mM Na3VO4, 10% glycerol, containing freshly added protease and phosphatase inhibitors (Roche Applied Science), and incubated in ice for 30 minutes. Cell lysates were collected by centrifugation at 13,000 x g for 15 minutes at 4°C. Total protein concentrations were measured using Bio-Rad DC protein assay kit (Bio-Rad), and adjusted to 1.5 µg/µl. 4X SDS Sample Buffer (40% Glycerol, 8% SDS, 0.25M Tris‐HCL, pH 6.8) plus β‐mercaptoethanol (1/10 of the volume) was added to a final volume of 120µl. Samples were boiled for 5 minutes, stored at -80°C, sent in dry ice to the RPPA Core facility, and processed as previously described [9,10].

**Purification of total RNA and Reverse Transcription-quantitative Polymerase Chain Reaction (RT-qPCR)**

Total RNA was extracted using Magmax 96 Total RNA isolation kit (Ambion) or RNeasy Mini Kit (Qiagen) according to the manufacturer’s instructions. cDNA was obtained from total RNA, previously treated with RQ1 RNase-free DNase (Promega), using OneScript Plus cDNA Synthesis Kit (Applied Biological Materials Inc. (abm), BC; Canada) or Superscript III reverse transcriptase (Invitrogen, Carlsbad, CA), following the manufacturer’s instructions. Quantitative PCR reactions were performed in 384-well plates with a Thermal iCycler (Bio-Rad Laboratories, Hercules, CA; USA) using the Bio-Rad iQ SYBR Green Supermix or BlasTaq™ 2X qPCR MasterMix (Applied Biological Materials Inc. (abm), BC; Canada) according to the manufacturer’s instructions. The PCR cycling conditions were as follows: 95°C for 10 minutes followed by 40 cycles at 95°C for 15 seconds and 60°C for 1 minute. The oligonucleotide primer pairs used for RT-qPCR were designed with PrimerBLAST (http://www.ncbi.nlm.nih.gov/tools/primer-blast/), and available upon request. To confirm the amplification specificity, the PCR products were subjected to the analysis of melting curve, linearity and slope of the standard curve. All PCR assays were performed in triplicate and the average Ct (cycles to threshold) was used for the comparative Ct method [11]. Quantification of GAPDH or HUPO levels served as an endogenous control. Control infections with scrambled shRNA, empty vectors, or non-infected cells were used to define 100% expression. The list of RT-qPCR primers used in the present study is reported in Additional Table S3.

**Western Blotting**

Protein extracts were prepared using Lysis Buffer containing 20 mM Tris-HCl (pH 7.4), 150 mM NaCl, 5 mM EDTA, 1% Triton X-100, 1 mM PMSF, 10 mM NaF, 1 mM Na3VO4, and Protease Inhibitor Cocktail (Roche, Basilea, Switzerland) and protease inhibitors (Roche, Mannheim, Germany) and incubated at 4°C for 30 minutes. Cell lysates were collected by centrifugation at 13,000 x g for 15 minutes. Total protein concentrations were measured using Bio-Rad DC protein assay kit (Bio-Rad). Equal amounts of protein lysates were resolved by SDS-PAGE, transferred to nitrocellulose membrane, blocked for 1 hour at room temperature with 5% low-fat milk in phosphate- buffered saline (PBS) solution with 0.1% Tween 20, and then incubated overnight with the primary antibodies, diluted in BSA 5% + NaN3, at room temperature. After 3 washes, membranes were incubated with the secondary antibody, diluted in PBT 5% low-fat milk, for 1 hour at room temperature. After 3 washes, the immune complexes were detected using Immobilon Western Chemiluminescent HRP Substrate (Merk). The list of antibodies used in the current study is reported in Additional Table S4.

**Analysis of apoptosis and cell cycle**

Apoptosis was measured by flow cytometry after staining with tetrametylrodamine methyl ester (TMRM; Molecular Probes, Eugene, Oregon, USA) or Annexin V-FITC Kit (Miltenyi Biotec, Bergisch Gladbach, Germany), according to the manufacturer's instructions. CD138^+^ cells were identified by anti hCD138-APC antibody (clone: 44F9; 130-117-395, Miltenyi Biotec). Cell cycle was measured by propidium iodide (PI) staining – flow cytometry. Briefly, cells were washed in PBS, treated with RNase (0.14 mg/mL) and incubated with propidium iodide (28.57 μg/mL). Data were acquired using BD FACSCelesta™ cytofluorimeter and processed with FACSDiva 8.0 software (BD Biosciences).

**ATPlite Assay**

ATPlite assay was performed using CellTiter-Glo® Luminescent Cell Viability Assay (Promega) to measure cell viability/proliferation. For each experiment cells were plated at a density of 1 x 10^5^/ml and cell proliferation was measured at day 0, day 3 and 4. 10μL of cells were mixed with an equal volume of CellTiter-Glo® Reagent solution per well in 384 - well white plate, in technical duplicate. Plate was shaken in the dark for 2 minutes and then incubated at room temperature for 10 minutes. Luminescence was measured using BioTek SynergyTM 2 Multi-Mode Microplate Reader (Bio-Tek Instruments, Winooski, Vermont, USA).

**Zebrafish housing**

Adult wild-type zebrafish (Danio Rerio; Tuebingen strain) were routinely maintained under a 14h light and 10h dark photoperiod at approximately 28°C, bred and genotyped according to standard procedures. Eggs were generated by natural mating, and following fertilization were collected, treated, and maintained under a12h light and 12h dark photoperiod at 28°C. Embryonic ages are expressed as hours post fertilization (hpf). Embryos were treated with 0.003% 1-phenyl-2-thiourea (PTU, #P7629, Sigma) at 24 hpf to prevent the formation of melanin pigment, which could interfere with the visualization of tumoral fluorescence injected cells. Adult fish were sacrificed with a tricaine overdose. Dechorionated zebrafish embryos (72 hpf) were anesthetized with 0.04 mg mL−1 tricaine (Sigma, St. Louis, MO, USA).

**Additional References**

[1] Besse L, Besse A, Mendez-Lopez M, Vasickova K, Sedlackova M, Vanhara P, et al. A metabolic switch in proteasome inhibitor-resistant multiple myeloma ensures higher mitochondrial metabolism, protein folding and sphingomyelin synthesis. Haematologica 2019;104:e415 LP-e419. https://doi.org/10.3324/haematol.2018.207704.

[2] Soriano GP, Besse L, Li N, Kraus M, Besse A, Meeuwenoord N, et al. Proteasome inhibitor-adapted myeloma cells are largely independent from proteasome activity and show complex proteomic changes, in particular in redox and energy metabolism. Leukemia 2016;30:2198–207. https://doi.org/10.1038/leu.2016.102.

[3] Maiques-Diaz A, Spencer GJ, Lynch JT, Ciceri F, Williams EL, Amaral FMR, et al. Enhancer Activation by Pharmacologic Displacement of LSD1 from GFI1 Induces Differentiation in Acute Myeloid Leukemia. Cell Rep 2018;22:3641–59. https://doi.org/10.1016/j.celrep.2018.03.012.

[4] Wiznerowicz M, Trono D. Conditional suppression of cellular genes: lentivirus vector-mediated drug-inducible RNA interference. J Virol 2003;77:8957–61. https://doi.org/10.1128/jvi.77.16.8957-8951.2003.

[5] Bergaggio E, Riganti C, Garaffo G, Vitale N, Mereu E, Bandini C, et al. IDH2 inhibition enhances proteasome inhibitor responsiveness in hematological malignancies. Blood 2019;133:156–67. https://doi.org/10.1182/blood-2018-05-850826.

[6] Gugnoni M, Manzotti G, Vitale E, Sauta E, Torricelli F, Reggiani F, et al. OVOL2 impairs RHO GTPase signaling to restrain mitosis and aggressiveness of Anaplastic Thyroid Cancer. J Exp Clin Cancer Res 2022;41:1–17. https://doi.org/10.1186/s13046-022-02316-2.

[7] Dobin A, Davis CA, Schlesinger F, Drenkow J, Zaleski C, Jha S, et al. STAR: Ultrafast universal RNA-seq aligner. Bioinformatics 2013;29:15–21. https://doi.org/10.1093/bioinformatics/bts635.

[8] Love MI, Huber W, Anders S. Moderated estimation of fold change and dispersion for RNA-seq data with DESeq2. Genome Biol 2014;15:1–21. https://doi.org/10.1186/s13059-014-0550-8.

[9] Tibes R, Qiu YH, Lu Y, Hennessy B, Andreeff M, Mills GB, et al. Reverse phase protein array: Validation of a novel proteomic technology and utility for analysis of primary leukemia specimens and hematopoietic stem cells. Mol Cancer Ther 2006;5:2512–21. https://doi.org/10.1158/1535-7163.MCT-06-0334.

[10] Cheng KW, Lu Y, Mills GB. Assay of Rab25 function in ovarian and breast cancers. Methods Enzymol 2005;403:202–15. https://doi.org/10.1016/S0076-6879(05)03017-X.

[11] Schmittgen TD, Livak KJ. Analyzing real-time PCR data by the comparative CT method. Nat Protoc 2008;3:1101–8. https://doi.org/10.1038/nprot.2008.73.

**Additional Figures and Legends**

**Figure S1.** **Loss-of-function screenings performed in PI-resistant MM cell lines.** (A) Experimental design of the shRNA screening or (B) drug screening performed in PI-resistant KMM-1 and U266 MM cell lines. (C) Representation of EOB value (x-axis) for all drug library compounds (y-axis). Data were ranked from more additive/synergistic (dotted red square) to antagonistic (dotted blue square). The top 15 candidates were selected using an arbitrary cut-off ≥ 0.2 on EOB in at least two concentrations. (D) Heatmap of the EOB values for each dilution of the top 15 candidates drugs found to be synergistic with CFZ, given at sublethal concentration (2,5 nM). EOB, excess over bliss.

**Figure S2. CoMMpass database investigation. LSD1 expression is increased in bortezomib-resistant MM patients and correlates to worse survival.** (A) MMRF CoMMpass dataset IA18 analysis showed a significant correlation between LSD1 expression (RNAseq, TPM) and Progression-free survival (N=123, P=0.0006). One standard deviation from the LSD1 mean expression was used to define the sub-populations as low (blue line) or high (red line) expression.

**Figure S3. Analysis of LSD1 silencing using constitutive and inducible shLSD1**. (A) KMM-1^PIR^ and (B) U266^PIR^ cells were transduced with lentiviral particles expressing two shRNA (shLSD1_D9, shLSD1_D10) targeting LSD1 or scramble shRNA (shCTRL). LSD1 silencing was monitored by RT-qPCR after puromycin selection. Data are the means ± s.d. of three independent experiments (C) Representative western blot showing LSD1 expression levels in KMM-1^PIR^ cells transduced with lentiviral particles expressing shCTRL or the indicated shLSD1. α-tubulin expression was included for protein loading normalization. (D-F) Western blot analysis showing LSD1 and GFP expression in KMS-28 TTA and (G-I) AMO-1 TTA cell lines transduced with lentiviral particles expressing indicated shLSD1 or shCTRL, upon induction with DOX. Cells were treated with 1µg/ml of DOX and pellets were collected at the indicated time points. α-tubulin protein expression was included for loading normalization. PIR, proteasome inhibitors resistant; RT-qPCR, quantitative real-time PCR; DOX, doxycycline; hrs, hours.

**Figure S4. LSD1 inhibitors testing.** (A-B) KMM-1^PIR^ and LP1 cell lines were treated with CFZ (10 nM and 1,25 nM respectively) and GSK2879552 (10 μM) or (B) GSK-LSD1 (10 μM). Cell viability was measured by TMRM staining-flow cytometry at 48 hpt (C) MOLM-13 cells were treated with SP2509, GSK2879552 and GSK-LSD1 at indicated concentrations. Cell cycle was measured by PI staining-flow cytometry 96 hpt. Blue, grey, and red bars represent G0/G1, S, and G2/M fractions respectively. (D) AMO-1 cells were treated with SP2509, GSK2879552 and GSK-LSD1 at the indicated concentrations. Cell cycle was measured by PI staining-flow cytometry 72 hpt. (E) KMM-1^PIR^, RPMI-8226^PIR^ and KMS-11 cell lines were treated with BTZ (5nM for KMS-11; 10 nM for KMM-1^PIR^; 300 nM for RPMI-8266^PIR^) in combination or not with SP2509 (100 nM for KMS-11 and KMM-1^PIR^; 2 µM for RPMI-8266^PIR^). Cell viability was estimated by TMRM staining-flow cytometry 72 hpt. (F) KMM-1^PIR^ and KMS-11 were treated with 50 nM and 20 nM of ixzazomib, respectively, and 100 nM SP2509. Cell viability was estimated by TMRM staining-flow cytometry 72- and 48- hpt. Data are the means ± s.d. of at least three independent experiments. (*P<.05; **P<.01; ***P<.001; ****P<.0001; ns>.05).

**Figure S5. SP2509/CFZ treatment is associated with antiproliferative and apoptotic programs.** (A) U266 cells were treated with CFZ (2,5 nM), SP2509 (1 µM), and the combinations. Cell viability was estimated by FACS (% of TMRM positive cells) at indicated time points. Data are the means ± s.d. of five independent experiments (***P<.001; ****P<.0001). (B) Venn diagram showing specific and commonly deregulated genes by CFZ, SP2509 and combo compared to DMSO. (C-E) Western blot analysis of indicated proteins in U266 cells treated with CFZ (2,5 nM), SP2509 (1 µM), or the combination for 24 hours. α-tubulin and vinculin were used for protein loading normalization.

**Figure S6. SP2509 but not GSK-LSD1 synergize with CFZ in MM cells.** (A) Dot plot graph of enriched GO terms from CFZ/SP2509 deregulated proteins in RPPA experiments performed in the U266 cell line treated with DMSO, CFZ (2,5 nM), SP2509 (1 µM) or the combination. The 9 GO processes with the largest gene ratios are plotted in order of fold enrichment. The size of the dots represents the number of genes in the significant DEG list associated with the GO term and the color of the dots represent the P-adjusted values. (B) Venn diagram showing specific and commonly deregulated genes between CFZ/SP2509 and CFZ/GSK-LSD1 combo treatments from RNA-Seq experiments. The rectangles below summarize the most enriched pathway, found performing GO analysis of BP process.

**Figure S7. CFZ/SP2509 combinatorial treatment is not toxic to normal cells.** (A) U266 in co-culture with HS-5 cells, U266 monoculture or HS-5 monoculture were treated with the DMSO, SP2509 (1 µM), CFZ (2,5 nM) or the combination. Cell viability was estimated by FACS (% of TMRM positive cells) 8 days post treatments. Data are the means ± s.d. of three independent experiments (***P<.001). (B) Body weight of NSG mice subcutaneously injected with KMS-28 TTA_shLSD1 and treated with vehicle (n=22), 4 mg/kg CFZ (n=19), 0,25 mg/mL DOXY (n=11), or a combination of both compounds (n=7).

**Additional Tables**

**Table S1. IC_50_ of PIR-MM cells used in the present study**

| CELL LINE | IC_50_ BTZ (nM) | IC_50_ CFZ (nM) | PSMB5 Status |
| --- | --- | --- | --- |
| KMM-1 | 7.846 | 3.215 | wt |
| U266 | 3.991 | 4.960 | N/A |
| AMO-1 | 4.009 | 4.798 | wt |
| RPMI-8226 | 5.259 | 10.81 | wt |
| U266^PIR^ | 20.096 | 39.643 | N/A |
| KMM-1^PIR^ | 22.164 | 89.966 | wt |
| AMO-1^PIR^ | 178.2 | 195.7 | wt |
| RPMI-8226^PIR^ | 167.4 | 253.6 | wt |

N/A not available

**Table S2. shRNA sequences used in the present study.**

| GENE_SYMBOL GENE_DESCRIPTION | GENE_ID | REFSEQ_ID | TRC_ID | OLIG_SEQ | CLONE_NAME | OLIG_ID |
| --- | --- | --- | --- | --- | --- | --- |
| MISSION pLKO.1-puro  Non-Mammalian shRNA  Control | N/A | N/A | N/A | CCGGCAACAAGATGAAGAGCACCAACTCGAGTTGGTGCTCTTCATCTTGTTGTTTTT | N/A | N/A |
| KDM1A lysine (K)-specific demethylase 1A | 23028 | NM_015013 | TRCN0000382379 | GTACCGGGGAGCTCCTGATTTGACAAAGCTCGAGCTTTGTCAAATCAGGAGCTCCTTTTTTG | NM_015013.3-2833s21c1 | D6 |
| KDM1A lysine (K)-specific demethylase 1A | 23028 | NM_015013 | TRCN0000046068 | CCGGGCCTAGACATTAAACTGAATACTCGAGTATTCAGTTTAATGTCTAGGCTTTTTG | NM_015013.1-1812s1c1 | D9 |
| KDM1A lysine (K)-specific demethylase 1A | 23028 | NM_015013 | TRCN0000046072 | CCGGCCACGAGTCAAACCTTTATTTCTCGAGAAATAAAGGTTTGACTCGTGGTTTTTG | NM_015013.1-1896s1c1 | D10 |

N/A not available

**Table S3. Primer sequences used in the present study. RT-qPCR: Reverse Transcription-quantitative Polymerase Chain Reaction.**

| Gene name | Primer sequence | Use |
| --- | --- | --- |
| LSD1 | Fw: 5’-AGCCATGGTGGTAACAGGTC-3’  Rv: 5’-AGCTTGTCCGTTGGCTTCATA-3’ | RT-qPCR |
| PLK1 | Fw: 5’-AAAGGGCACAGTTTCGAGGT-3’  Rv: 5’-AGGTGGTTTGCCCACTAACA-3’ | RT-qPCR |
| AURKA | Fw: 5’-CCTGAGGAGGAACTGGCATC-3’  Rv: 5’-CCAGAGGGCGACCAATTTCA-3’ | RT-qPCR |
| IRF4 | Fw: 5’-GGCAAGCAGGACTACAACCG-3’  Rv: 5’-TTGTCGATGCCTTCTCGGAAC-3’ | RT-qPCR |
| KLF2 | Fw: 5’-CACACAGGTGAGAAGCCCTACC-3’  Rv: 5’-CTTTCGGTAGTGGCGCGTG-3’ | RT-qPCR |
| H2AC7 | Fw: 5’-GCTGTACTGCTCCCCAAGAA-3’  Rv: 5’-GAGCCTTTGTTAAGACTGCTTCC-3’ | RT-qPCR |
| H2AC14 | Fw: 5’-GCAAAGTCACCATCGCACAG-3’  Rv: 5’-ATGCGCTTTTCAACCGGTC-3’ | RT-qPCR |
| H2BC14 | Fw: 5’-CTGCTACCCGGGGAATTGG-3’  Rv: 5’-GGTCACGGCGGAACTGTTA-3’ | RT-qPCR |
| H4C9 | Fw: 5’-CCATGGACGTGGTCTACGC-3’  Rv: 5’-GACTGGGCTTCAAAATGCCA-3’ | RT-qPCR |
| H2AC4 | Fw: 5’-AGGGTGGCGTTTTGCCTAAT-3’  Rv: 5’-AAACAGCAGTGCATGAAGCG-3’ | RT-qPCR |
| H3C12 | Fw: 5’-CTGTGCTATTCACGCCAAGC-3’  Rv: 5’-ACCCAAGATAGAGCAGGGGA-3’ | RT-qPCR |
| H1-3 | Fw: 5’-GTGGCCAAGAGTGCGAAAAA-3’  Rv: 5’-CTTCCCCGACTTAGGCTTGG-3’ | RT-qPCR |
| HUPO | Fw: 5’-GCTTCCTGGAGGGTGTCC-3’  Rv: 5’-GGACTCGTTTGTACCCGTTG-3’ | RT-qPCR |

**Table S4. Antibodies used in the present study.**

| **Antibody** | **Spieces** | **Clone, Cat. Num.** | **Company** |
| --- | --- | --- | --- |
| α-tubulin | mouse | B-5-1-2, T5168 | Sigma-Aldrich |
| vinculin | mouse | SAB4200080 | Sigma-Aldrich |
| α-Tubulin | mouse | B-5-1-2, sc-23948 | Santa Cruz Biotechnology |
| β-Actin | mouse | AC-15, sc-69879 | Santa Cruz Biotechnology |
| cyclin E | mouse | HE12; sc-247 | Santa Cruz Biotechnology |
| cyclin A | rabbit | H-432; sc-751 | Santa Cruz Biotechnology |
| cyclin B1 | rabbit | H433; sc-752 | Santa Cruz Biotechnology |
| PARP-1 | rabbit | H-250; sc-7150 | Santa Cruz Biotechnology |
| p21 | mouse | 610233 | BD Biosciences |
| Bcl-2 | mouse | M0887 | Agilent Dako |
| cleaved PARP-1 | rabbit | #5625 | Cell Signaling Technology |
| cleaved caspase-9 | rabbit | #7237 | Cell Signaling Technology |
| cleaved caspase-7 | rabbit | #8438 | Cell Signaling Technology |
| cleaved caspase-3 | rabbit | #9664 | Cell Signaling Technology |
| LSD1 | rabbit | C69G12, #2184 | Cell Signaling Technology |
| GFP | rabbit | #2555 | Cell Signaling Technology |
| Myc-Tag | mouse | 9B11, #2276 | Cell Signaling Technology |
| PLK1 | rabbit | 208G4, #4513 | Cell Signaling Technology |
| XIAP | rabbit | 3B6, #2045 | Cell Signaling Technology |
| Bcl-xL | rabbit | 54H6, #2764 | Cell Signaling Technology |
| c-IAP1 | rabbit | D5G9, #7065 | Cell Signaling Technology |
| Mcl-1 | rabbit | #4572 | Cell Signaling Technology |
| p-Chk2 (T68) | rabbit | C13C1, #2197 | Cell Signaling Technology |
| Chk2 | mouse | 1C12, #3440 | Cell Signaling Technology |
| p-Chk1 (S345) | rabbit | 133D3, #2348 | Cell Signaling Technology |
| Chk1 | mouse | 2G1D5, #2360 | Cell Signaling Technology |
| p-p53 (S15) | rabbit | 16G8, #9286 | Cell Signaling Technology |
| p53 | rabbit | #9282 | Cell Signaling Technology |
| p-BRCA1 (S1524 | rabbit | #9009 | Cell Signaling Technology |
| BRCA1 | rabbit | (#9010 | Cell Signaling Technology |
| Histone H2A.X (S139) | rabbit | 20E3, #9718 | Cell Signaling Technology |
| p-ATM (S1981) | rabbit | D6H9, #5883 | Cell Signaling Technology |
| ATM | rabbit | #2873 | Cell Signaling Technology |
| Mono-Methyl-Histone H3 (K4) | rabbit | D1A9, #5326 | Cell Signaling Technology |
| Di-Methyl-Histone H3 (K4) | rabbit | C54G9, #9725 | Cell Signaling Technology |
| Mono-Methyl-Histone H3 (Lys9) | rabbit | D1P5R, #14186 | Cell Signaling Technology |
| Di-Methyl-Histone H3 (Lys9) | rabbit | D85B4, #4658 | Cell Signaling Technology |
| Histone H3 | rabbit | D1H2, #4499 | Cell Signaling Technology |
| p-AURORA_A/B/C (T288/232/198) | rabbit | D13A11, #2914 | Cell Signaling Technology |
| Aurora A | rabbit | D3E4Q, #14475 | Cell Signaling Technology |
